# Inhibition of Microglial TRPV1 Ameliorates Brain Injury After Intracerebral Hemorrhage by Suppressing AMPK/PINK1‐Mediated Mitophagy

**DOI:** 10.1002/cns.70881

**Published:** 2026-04-20

**Authors:** Kezhu Chen, Xiangyang Deng, Jun Zeng, Baoye Sun, Jingyu Yu, Tianwen Li, Junjie Zhong, Pengjie Hong, Peng Wang, Fengshi Li, Quan Zhang, Junwei Ren, Qisheng Tang, Tongming Zhu, Jianhong Zhu

**Affiliations:** ^1^ Department of Neurosurgery, Huashan Hospital, Shanghai Medical College Fudan University Shanghai China; ^2^ National Center for Neurological Disorders Shanghai China; ^3^ National Clinical Center for Geriatric Disorders, Fudan University Shanghai China; ^4^ State Key Laboratory of Brain Function and Disorders Shanghai China; ^5^ Shanghai Key Laboratory of Brain Function and Regeneration Shanghai China; ^6^ Institute of Neurosurgery Fudan University Shanghai China; ^7^ Institutes of Brain Science Fudan University Shanghai China; ^8^ Ministry of Education Frontier Science Center for Brain Science Fudan University Shanghai China; ^9^ Department of Neurosurgery The First Affiliated Hospital of Chongqing Medical University Chongqing China; ^10^ Department of Liver Surgery and Transplantation, Zhongshan Hospital Fudan University Shanghai China

**Keywords:** capsaicin, capsazepine, intracerebral hemorrhage, mitophagy, TRPV1

## Abstract

**Background:**

The transient receptor potential vanilloid 1 (TRPV1) is a cation channel implicated in neurological disorders. Although TRPV1 activation contributes to intracerebral hemorrhage (ICH) pathology, its microglia‐specific role and underlying mechanisms remain poorly defined. This study investigates how microglial TRPV1 influences ICH injury.

**Methods:**

We utilized a mouse ICH model alongside microglia‐specific TRPV1 knockout mice, BV2 cells, and primary microglial cultures. Interventions included TRPV1 antagonist capsazepine (CPZ), agonist capsaicin (CAP), microglial depletion agent PLX5622, and TRPV1 knockdown. Outcomes were assessed using immunofluorescence, behavioral tests, Western blot, magnetic resonance imaging (MRI), and transmission electron microscopy (TEM).

**Results:**

TRPV1 expression was significantly upregulated post‐ICH, primarily in microglia. TRPV1 blockade with CPZ treatment reduced hematoma volume, brain edema, neuronal apoptosis, and improved neurological function, whereas CAP exacerbated injury. These benefits were replicated in microglia‐specific TRPV1 knockout mice. Mechanistically, CPZ shifted microglia from a pro‐inflammatory (iNOS+) to a regulatory (Arg1+) phenotype and suppressed excessive mitophagy via the Ca^2+^‐AMPK‐PINK1 pathway.

**Conclusion:**

TRPV1 activation in microglia exacerbates ICH injury by promoting inflammation and disruptive mitophagy. Targeted inhibition of microglial TRPV1 represents a promising therapeutic strategy for ICH.

## Graphical abstract

After intracerebral hemorrhage, microglial TRPV1 channel opening increases intracellular calcium, triggering excessive AMPK‐PINK1‐mediated mitophagy. This causes microglial dysfunction, neuronal necrosis/apoptosis, blood–brain barrier disruption, and neurological impairment. Inhibiting microglial TRPV1 (via inhibitors or conditional knockout) promotes the transformation of activated microglia into regulatory phenotype, reduces mitophagy, and facilitates recovery.

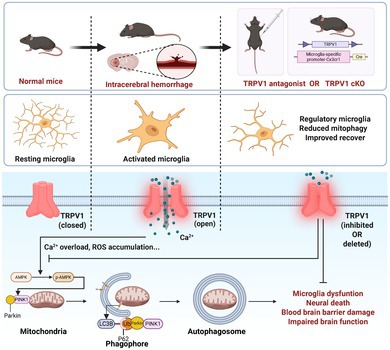

**Abbreviations**
BBB: blood–brain barrier
CAP: capsaicin
cKO: conditional knockout
CPZ: capsazepine
DG: dentate gyrus
DMSO: dimethylsulfoxide
ECL: enhanced chemiluminescence
FJC: Fluoro‐Jade C
FP test: forelimb placement test
Hb: hemoglobin
HBSS: hanks balanced salt solution
ICH: intracerebral hemorrhage
mNSS: modified neurological deficit score
MRI: magnetic resonance imaging
OCT: optimal cutting temperature
PBS: phosphate‐buffered saline
PFA: paraformaldehyde
SD: standard deviation
TEM: transmission electron microscope
TRPV1: transient receptor potential vanilloid 1
TUNEL: terminal deoxynucleotidyl transferase dUTP nick end labeling

## 1 Introduction

ICH is a devastating stroke subtype with rapid onset, high mortality, and substantial societal burden [1]. Its pathology involves not only the primary mechanical injury from vessel rupture but also secondary damage mechanisms such as microglial activation, iron overload, and oxidative stress [2, 3].

The TRPV1 channel, activated by various lipophilic compounds and physical stimuli such as heat and osmotic changes, is increasingly recognized for roles beyond pain perception, including in immune response and cell growth [4, 5, 6]. In the central nervous system, TRPV1 is enriched in microglia and is a key mediator of microglia–neuron communication and neuroinflammation [7, 8]. While TRPV1 overactivation can provoke mitochondrial damage and cell death [9], its inhibition has been shown to attenuate microglial reactive oxygen species production and reduce neuronal apoptosis in ICH models [10, 11]. These findings highlight TRPV1's significant yet incompletely understood role in ICH pathophysiology.

A recent study by Chen et al. demonstrated that pharmacological inhibition or global knockout of TRPV1 reduces brain damage and neuronal apoptosis after ICH [11]. However, a critical question remained unanswered: through which cell type does TRPV1 exert its detrimental effects? TRPV1 is expressed in multiple CNS cell types, including neurons, microglia, and vascular cells [4, 7]. Given that microglial activation is a hallmark of ICH pathology and critically influences disease progression [12], the specific function of TRPV1 in regulating microglia following ICH requires further elucidation.

Mitochondrial function is essential for microglial homeostasis. Microglia require functional mitochondria to meet the high energy demands of phagocytosis and cytokine production after ICH [13, 14]. Meanwhile, TRPV1 is intimately linked to mitochondrial regulation. TRPV1 activation induces calcium influx, which can trigger mitochondrial damage and autophagy [9, 15]; conversely, TRPV1 inhibition preserves mitochondrial function in various disease models [16, 17, 18]. It remains unclear how the activity of TRPV1 in ICH affects mitochondria and the outcome of ICH.

Meanwhile, the role of autophagy in ICH is highly controversial. Some studies suggest a protective function, whereas others indicate that heightened microglial autophagy exacerbates neuroinflammation and worsens outcomes [19, 20]. The relationship between TRPV1 and autophagy is also context‐dependent. TRPV1 activation can induce oxidative stress‐triggered microglial autophagy and apoptosis under conditions such as oxygen–glucose deprivation [15, 21], yet it has also been linked to protective autophagy in neurodegenerative settings [22, 23]. Emerging evidence suggests that the relationship between autophagic activity and cellular function follows a “U‐shaped” curve, wherein moderate activation supports homeostasis but excessive activation becomes detrimental [24, 25]. Moderate mitophagy—autophagy targeting mitochondria—might help resolve this paradox by maintaining mitochondrial quality control in the oxidative stress‐rich ICH environment. Thus, whether microglia‐specific inhibition of TRPV1 could attenuate excessive mitophagy, preserve mitochondrial integrity, and improve ICH outcomes needs to be clarified. In this study, we characterized the expression pattern of TRPV1 after ICH and evaluated the consequences of its pharmacological and genetic modulation. Combining in vivo and in vitro approaches, we identified microglia as the primary cellular mediator of TRPV1 effects in ICH, and established that TRPV1 influences ICH outcomes by regulating microglial autophagy.

## 2 Material and Methods

### 2.1 Animals

Male C57BL/6 mice (8–10 weeks old, 25–30 g) were obtained from Vital River Laboratory Animal Co. Ltd. All mice were housed under SPF conditions with a controlled 12‐h light/dark cycle, constant temperature and humidity, and free access to food and water. TRPV1^*flox/flox*^; Cx3cr1^*Cre*^ conditional knockout (cKO) mice were generated by crossing TRPV1^*flox/flox*^ mice with Cx3cr1^*Cre*^ mice (both from Shanghai Model Organisms Center Inc.). TRPV1^*flox/flox*^ littermates served as controls. Genotyping was performed by PCR using the following primers: TRPV1 flox (F: ATGGGGTGGGTGATGCTATGTGAC; R: AGCCATGGGTGCTGCTAAAT) and Cre (F: CAAGCGCTGTTGGTGAGAGA; R: GGATTCTCCTCGACGTCACC). All procedures were approved by the Animal Care Committee of Laboratory Animal Center Fudan University (Approval ID: 202509048Z) and followed ARRIVE guidelines.

### 2.2 Experimental Design

The experimental design was depicted in Figure S1.

### 2.3 ICH Model

The ICH model was established by stereotaxic injection of collagenase IV (0.0375 U in 0.5 μL saline) into the right striatum (coordinates: 0.5 mm anterior, 2.2 mm lateral to bregma, 3.5 mm depth) [26] under pentobarbital sodium anesthesia (0.3%, 0.1–0.2 mL/10 g). Sham‐operated mice underwent needle insertion without collagenase administration.

### 2.4 Drug Administration

Mice were randomly assigned to different groups and received first injection 1 h after ICH induction, followed by once‐daily injections for three consecutive days. The doses of CAP (5 mg/kg) and CPZ (1 mg/kg) were selected based on previous studies [27, 28].

### 2.5 Neurobehavioral Tests

Short‐term neurological function was assessed using the modified Neurological Severity Score (mNSS), forelimb placement test, and corner turning test [29]. Medium‐ and long‐term functions were evaluated with the open field test (Day 14) and the Morris water maze (Day 28).

### 2.6 Hematoma Volume and Hemoglobin Content

Hematoma volume and hemoglobin content were quantified 3 days post‐ICH using established methods [30]. The relative Hb levels are expressed as values normalized to the sham group (set as 1).

### 2.7 Brain Water Content Test

Brain water content was measured by the wet/dry weight method. Brain tissues were separated into ipsilateral and contralateral hemispheres and cerebellum. Each sample was weighed immediately to obtain wet weight, then dried at 100°C for 72 h and reweighed for dry weight. The brain water content was calculated as [(wet weight − dry weight)/wet weight] × 100%.

### 2.8 MRI

The lesion volume was evaluated via an 11.7 T small‐animal MRI apparatus (Bruker Corp.). The following setup parameters were used to acquire T2‐weighted images: repetition time, 2500 ms; image matrix, 256 × 256; echo time, 26 ms; in‐plane resolution, 0.1 × 0.1 mm^2^; field of view, 25.6 × 25.6 mm^2^; and slice thickness, 0.4 mm.

### 2.9 TEM

The mice were transcardially perfused with 2.5% glutaraldehyde. Subsequently, striatal samples (1 mm^3^) were immersion‐fixed in the same solution at 4°C for 12 h. After fixation, tissues were treated with 1% osmium tetroxide, dehydrated in ethanol, and embedded in epoxy resin. Ultrathin sections (70–90 nm) were prepared using a Leica EM UC7 ultramicrotome, contrasted with uranyl acetate and lead citrate, and visualized using a HITACHI HT7800 TEM. For quantification of mitophagosomes and damaged mitochondria, all TEM images were captured and analyzed by two independent investigators who were blinded to the experimental groups. And the average counts were used for statistical analysis.

### 2.10 Western Blot

Total protein was extracted from tissues or cells using a commercial kit (SD‐001/SN‐002, Invent Biotechnologies). Equal amounts of protein were separated by SDS‐PAGE and transferred to nitrocellulose membranes (Millipore). After blocking with 5% nonfat milk for 1 h, the membranes were incubated overnight at 4°C with primary antibodies (see Table S1), followed by incubation with species‐matched secondary antibodies (Cell Signaling Technology, 1:5000) for 2 h at room temperature. Protein bands were visualized using an enhanced chemiluminescence (ECL) substrate.

### 2.11 TUNEL Staining

A terminal deoxynucleotidyl transferase dUTP nick end labeling (TUNEL) staining kit (Roche, 11684795910) was used to identify in situ cell apoptosis in the peri‐hematoma area.

### 2.12 Immunofluorescence Staining

After euthanasia, mice were perfused transcardially with PBS followed by 4% paraformaldehyde (PFA). The harvested brains were postfixed in 4% PFA for 24 h, cryoprotected in 30% sucrose, and sectioned coronally (30 μm) using a Leica CM1950 microtome. Sections were permeabilized with 1% Triton X‐100 (20 min) and blocked with 5% BSA (1 h), after which they were incubated with primary antibodies (Table S2) overnight at 4°C. Following PBS washes, sections were incubated with corresponding secondary antibodies (Table S2) for 2 h at room temperature, and finally mounted with an antifade medium (Beyotime Biotechnology, P0131) containing DAPI.

### 2.13 FJC Staining

Three days after ICH, degenerative neurons were identified using a Fluoro‐Jade C (FJC) ready‐to‐dilute staining kit (Millipore). Briefly, sections were dried at 37°C for 2–3 days, followed by sequential incubation in solutions A, B, and C. They were then heated at 50°C for 10 min and finally cleared in xylene for 2 min.

### 2.14 Nissl Staining

After PBS rinses, brain sections were incubated in Nissl's stain reagent (Servicebio) for 3 min, briefly rinsed with distilled water, air‐dried at 65°C, and coverslipped with neutral resin.

### 2.15 Cell Culture

BV2 and HT22 cell lines (Pricella Biotechnology) were cultured in high‐glucose DMEM (Gibco) containing 10% FBS (Gibco) and 1% penicillin/streptomycin at 37°C in a 5% CO_2_ incubator. All experiments utilized cells between passages 10–30. For the in vitro ICH model, cells were exposed to 10 μM hemoglobin (Hb; Sigma) for 24 h. When applicable, the AMPK activator A769662 (30 μM) or inhibitor BAY‐3827 (1 μM) was added to the culture medium.

### 2.16 Primary Microglia Isolation

The mice were anesthetized and intracardially perfused with cold PBS. Then brains were removed, placed in ice‐cold HBSS, and dissected to remove the cerebellum, meninges, blood vessels, and choroid plexus. The tissue was then minced, digested with 0.25% trypsin at 37°C for 5 min. After filtering through a 70‐μm strainer and centrifugation, the pellet was resuspended in 37% isotonic Percoll and separated on a discontinuous Percoll gradient (70%/37%/30%) by centrifugation at 300 g for 30 min at 18°C (low acceleration/brake). Microglia were collected from the 37%/70% interface, washed with PBS, and cultured in complete DMEM on PDL‐coated plates.

### 2.17 Transfection of siRNA

siRNAs targeting human TRPV1 (siTRPV1) and scrambled siRNA were synthesized by GenePharma Biotech (Shanghai, China). TRPV1 siRNAs (Table S3) were transfected into BV2 cells via Lipofectamine 3000 transfection reagent (Invitrogen).

### 2.18 JC‐1 Staining

HT22 cells were stained with JC‐1 working solution (Beyotime Biotechnology, C2003S) at 37°C for 20 min to assess mitochondrial membrane potential, following the kit protocol.

### 2.19 Calcium Assay

BV2 cells were washed twice with HBSS and then loaded with 2 μM Rhod‐2/AM (Ca^2+^ probe; 40776ES72, Yeasen) for 30 min. Subsequently, the cells were washed three times with HBSS and further incubated in HBSS for 30 min at 37°C.

### 2.20 Mitochondria Extraction

The mitochondria and cytoplasm components in BV2 cells were extracted using the mitochondrial isolation kit (Beyotime, C3601), and then the subsequent experiments were conducted. The extraction method should follow the instructions provided by the manufacturer.

### 2.21 Phagocytosis Experiment

Blood was collected from the mice and red blood cells were separated by gradient centrifugation. Follow the manufacturer's instructions to incubate red blood cells with the PKH26 reagent (Beyotime, C2071S). Then, add the red blood cells to the BV2 cell culture medium and co‐culture them for 12 h.

### 2.22 Statistical Analysis

Statistical analyses were performed using GraphPad Prism 9. All data were presented as mean ± SD. Comparisons between two groups were made using Student's *t*‐test, while one‐way or two‐way ANOVA followed by Tukey's post hoc test was applied for multi‐group comparisons. Kruskal–Wallis test followed by Dunn's post hoc test was used to analyze the mNSS score. A *p*‐value of less than 0.05 was considered statistically significant.

## 3 Results

### 3.1 TRPV1 Expression Is Upregulated and Predominantly Localized in Microglia After ICH

**FIGURE 1:**
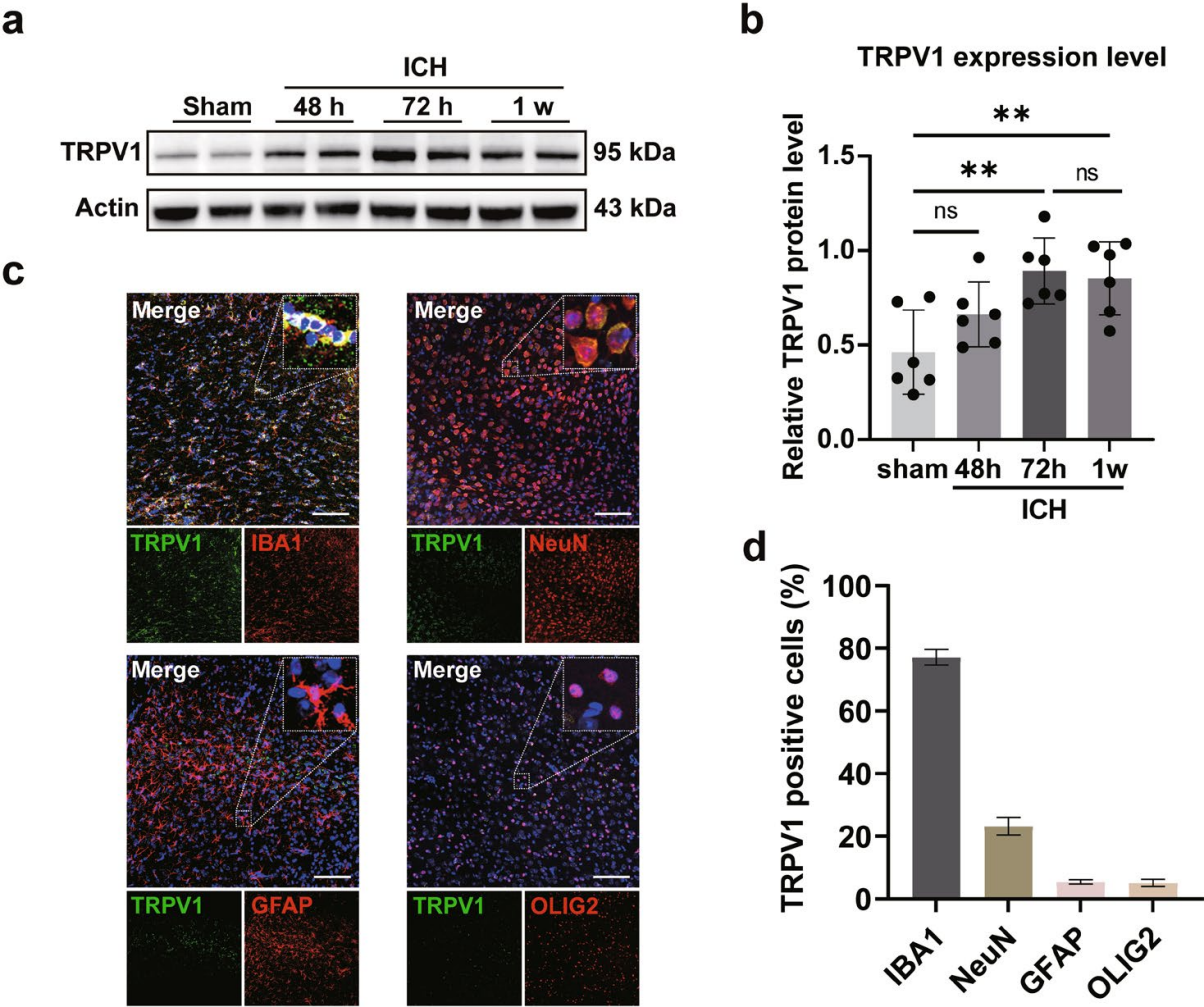
Expression pattern and cellular distribution of TRPV1 after ICH. (a and b) Western blot images and quantitative analysis of TRPV1 protein levels at different timepoints post‐ICH. Data were expressed as mean ± SD. **p < 0.01 vs. Sham, *n* = 6/group. (c) Images displaying the co‐staining of TRPV1 receptor (green) with microglia (IBA1, red), neurons (NeuN, red), astrocytes (GFAP, red), or oligodendrocytes (OLIG2, red) in the perihematomal area of mice 3 days post‐ICH. Scale bar is 100 μm, *n* = 4–6/group. (d) Statistical results of the proportion of TRPV1 receptor expression in microglia, neurons, astrocytes, and oligodendrocytes in the perihematomal area.

While TRPV1 is implicated in brain ischemia–reperfusion injury [31], its role in intracerebral hemorrhage (ICH) requires further investigation. We assessed TRPV1 protein levels around the hemorrhage site over time. Western blot analysis showed that TRPV1 expression was significantly upregulated on Day 3 post‐ICH (*p* < 0.05, Figure 1b) and remained elevated for 1 week. Immunofluorescence co‐staining revealed that TRPV1 was predominantly expressed in IBA1‐positive microglia, and to a lesser extent in NeuN‐positive neurons, with minimal expression in GFAP‐positive astrocytes or OLIG2‐positive oligodendrocytes (Figure 1c,d). Given the pivotal role of microglia in hematoma clearance and their accumulation around hemorrhagic areas, these findings suggest a potential role for TRPV1 in ICH pathogenesis, particularly within microglia.

### 3.2 Blocking of TRPV1 Promoted Hematoma Resorption and Alleviated Brain Edema

The effects of CAP on target cells depend on dose [32] and the degree of TRPV1 activation relative to disease severity. To determine optimal dosing, ICH mice received intraperitoneal injections of CAP (0.1, 0.5, or 1 mg/kg) or CPZ (0.5, 1.0, or 2 mg/kg). All CAP doses significantly improved the mNSS 1 week after ICH (*p* < 0.05, Figure S2), with 0.5 mg/kg showing efficacy comparable to 1 mg/kg; higher doses were avoided due to potential toxicity. While 0.5 mg/kg CPZ was ineffective (*p* > 0.05, Figure S2), both 1.0 and 2 mg/kg CPZ were beneficial (*p* < 0.05, Figure S2), with no difference between them. Consequently, 0.5 mg/kg CAP and 1 mg/kg CPZ were selected for subsequent studies. Although systemic TRPV1 modulation can affect the broader immune response [33], we chose intraperitoneal injection for its clinical translational feasibility.

**FIGURE 2:**
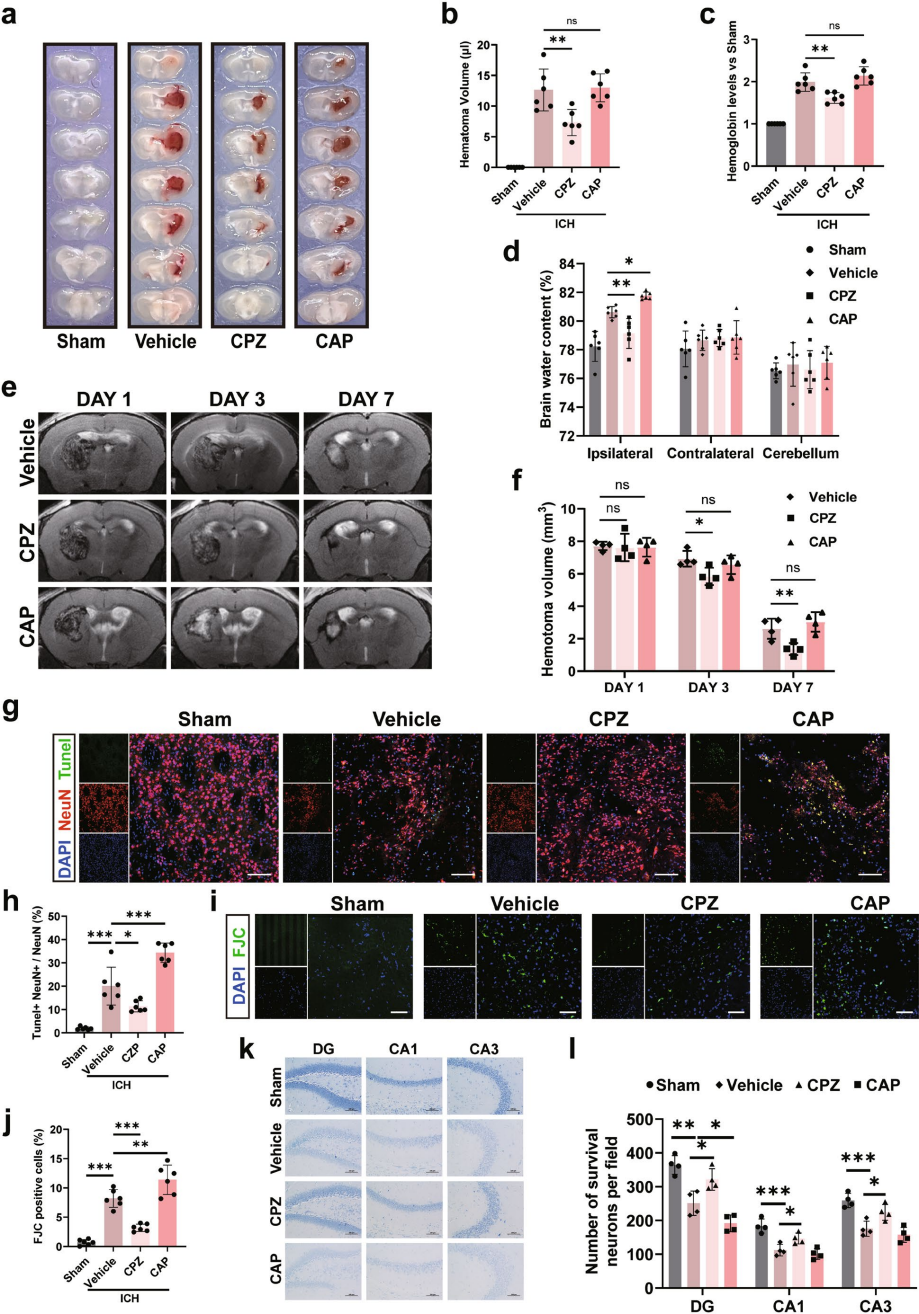
The effects of CPZ and CAP on hematoma absorption and neuronal survival after ICH. (a) Coronal brain sections 3 days post‐ICH. (b and c) Quantitative analysis of hematoma volume and hemoglobin levels in different groups. (d) Quantitative analysis of brain water content 3 days post‐ICH. (e) MRI images 1, 3, and 7 days after ICH. (f) Comparative analysis of hematoma volumes measured by MRI. (g and h) Images and quantitative analysis of TUNEL (green) and NeuN (red) colocalization in neurons within the perihematomal area, 3 days post‐ICH. (i and j) Images and quantitative analysis of FJC staining 3 days after ICH. Scale bar is 100 μm. (k) Nissl staining images of the hippocampus 28 days post‐ICH. Scale bar is 100 μm. (l) Quantification of surviving neurons per field, as indicated by Nissl staining. Data were expressed as mean ± SD. *p < 0.05, **p < 0.01, ***p < 0.001 vs. Vehicle. *n* = 6/group in a‐d and g‐j, *n* = 4/group in (e and f) and (k and l).

Treatment with CPZ significantly reduced hematoma volume on Day 3 post‐ICH (*p* < 0.05, Figure 2a,b) and decreased ipsilateral brain water content (*p* < 0.05, Figure 2d). In contrast, CAP did not affect hematoma volume (*p* > 0.05, Figure 2a,b) but worsened cerebral edema (*p* < 0.05, Figure 2d). Hemoglobin levels on Day 3 were consistent with the hematoma volume data (Figure 2c). No significant differences were observed in the contralateral hemisphere or cerebellum among groups (*p* > 0.05, Figure 2d).

Widespread blood–brain barrier (BBB) disruption due to peri‐hematomal vascular inflammation is a known consequence of ICH [34]. Consistent with this, CPZ treatment significantly upregulated the tight junction proteins ZO‐1 (*p* < 0.05, Figure S3a,b) and claudin‐5 (*p* < 0.05, Figure S3c,d) compared to the vehicle group at 3 days post‐ICH. This suggests that CPZ may ameliorate cerebral edema by restoring BBB integrity.

We also monitored hematoma volume in vivo using an 11.7 T high‐resolution MRI system. Initial hematoma volumes were comparable across all groups (*p* > 0.05, Figure 2e,f). Subsequent imaging revealed that CPZ treatment significantly reduced hematoma volume (*p* < 0.05, Figure 2e,f), whereas CAP had no significant effect compared to the vehicle group (*p* > 0.05, Figure 2e,f).

### 3.3 TRPV1 Blockade Alleviated Neuronal Apoptosis and Promoted Neuronal Survival After ICH

We next asked whether TRPV1 blockade confers neuroprotection after ICH. TUNEL and FJC staining revealed a marked increase in apoptotic and degenerating neurons after ICH (*p* < 0.05, Figure 2g,h). CPZ administration reduced the number of TUNEL‐positive neurons by approximately 50% (*p* < 0.05, Figure 2g,h), a finding consistent with reports in TRPV1 knockout mice [11]. Conversely, CAP treatment aggravated neuronal apoptosis (*p* < 0.05, Figure 2g,h). Consistent with the TUNEL data, CPZ also significantly decreased the number of degenerating FJC‐positive neurons (*p* < 0.05, Figure 2i,j). These data demonstrate that TRPV1 inhibition effectively mitigates ICH‐induced neuronal damage.

To evaluate long‐term neuronal damage, we performed Nissl staining on hippocampal tissues 4 weeks after ICH. CPZ‐treated mice showed significantly greater neuronal survival in the DG, CA1, and CA3 regions relative to the vehicle group (*p* < 0.05, Figure 2k,l). Conversely, CAP treatment resulted in significant neuronal loss restricted to the DG region (*p* < 0.05, Figure 2k,l), whereas other subfields were unaffected (*p* > 0.05, Figure 2k,l). These findings demonstrate that TRPV1 inhibition effectively mitigates neuronal loss following ICH.

### 3.4 TRPV1 Blockade With CPZ Ameliorates Short‐ and Long‐Term Neurological Deficits After ICH

To determine whether the neuroprotective and hematoma‐resolving effects of CPZ translate to improved functional outcomes, we subjected mice to a series of behavioral tests. The CAP‐treated group was excluded from these and subsequent experiments due to its consistent detrimental effects on hematoma resorption, neuronal survival (Figure 2), and early neurological function (Figure S2).

**FIGURE 3:**
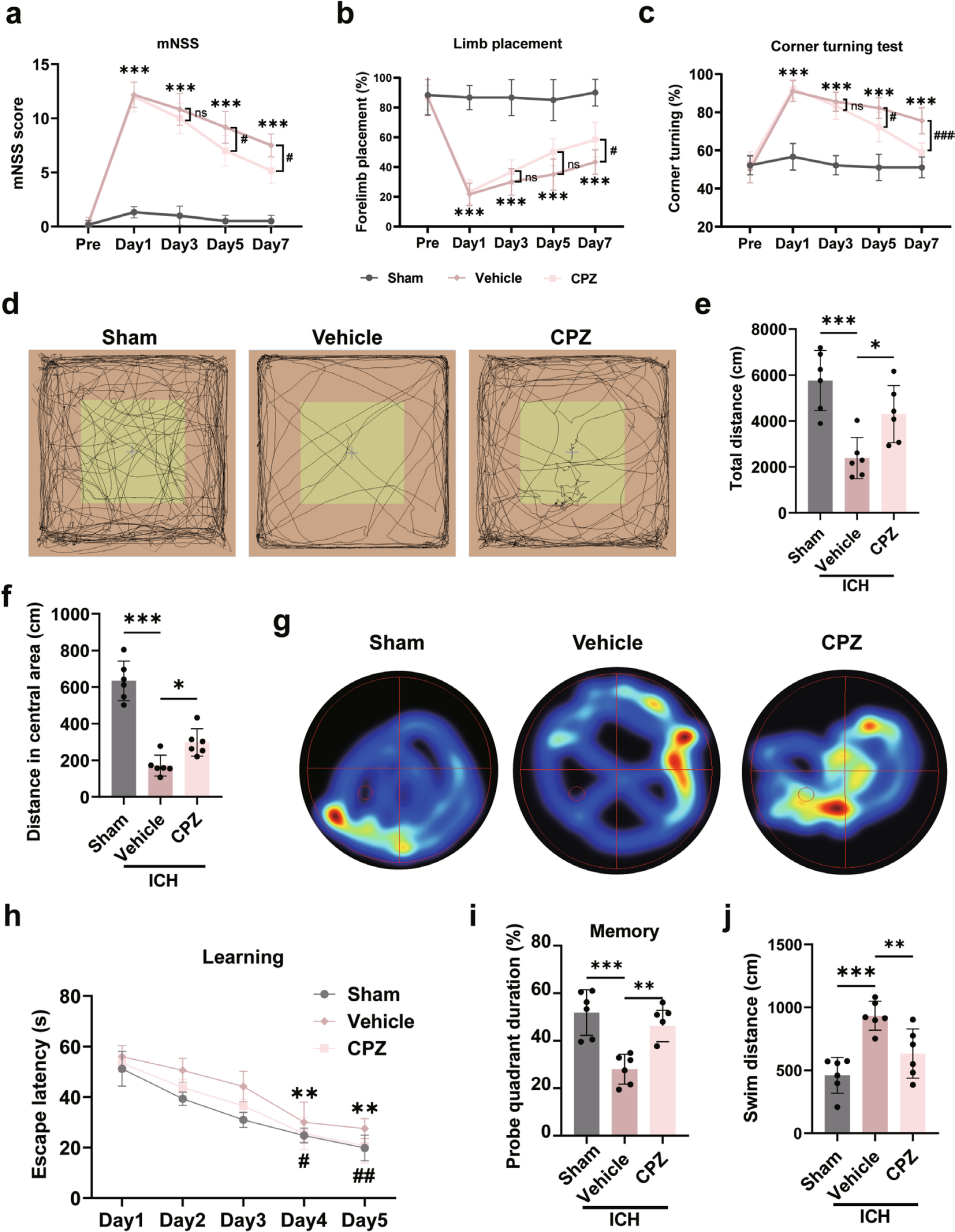
CPZ treatment improved both short‐ and long‐term neurobehavioral outcomes. (a–c) Modified neurological deficit score (mNSS), forelimb placement (FP) test, and corner turning test 1, 3, 5, and 7 days after ICH. (d) Motion trajectories in the open field test conducted 14 days after ICH. (e and f) Analysis of the total distance and distance in the central area in the open field test. (g) Heatmaps from the Morris water maze test 28 days post‐ICH. (h–j) Escape latency, probe quadrant duration, and swim distance in the Morris water maze test. Data were expressed as mean ± SD. *p < 0.05, **p < 0.01, ***p < 0.001 CPZ vs. Vehicle; ^#^ *p* < 0.05, ^##^ *p* < 0.01, ^###^ *p* < 0.001 sham vs. vehicle. *n* = 6/group.

CPZ treatment significantly improved short‐term neurological function after ICH across multiple behavioral assays. Compared to the vehicle group, CPZ administration reduced mNSS scores on Days 5 and 7 (*p* < 0.05, Figure 3a), increased the success rate in the foot‐fault test on Day 7 (*p* < 0.05, Figure 3b), and improved cornering test scores on Days 5 and 7 (*p* < 0.05, Figure 3c). Notably, cornering test performance in the CPZ group recovered to a level comparable with the sham group (*p* > 0.05, Figure 3c).

To evaluate long‐term neurological function, the open field test was performed 14 days post‐ICH. CPZ‐treated mice exhibited significant increases in total travel distance and central zone distance compared to the vehicle group (*p* < 0.05, Figure 3d–f). Furthermore, in the Morris water maze test beginning on Day 28, CPZ treatment improved spatial learning and memory. During acquisition, CPZ‐treated mice located the hidden platform faster on Days 4 and 5 (*p* < 0.05, Figure 3g,h), despite persistent deficits in the ICH model relative to sham controls (*p* < 0.05, Figure 3h). In the probe trial, they also spent more time in the target quadrant and had a shorter platform location distance than vehicle‐treated mice (*p* < 0.05, Figure 3i,j), indicating that TRPV1 blockade ameliorates ICH‐induced cognitive deficits.

### 3.5 Function of TRPV1 in ICH Is Microglia Dependent

**FIGURE 4:**
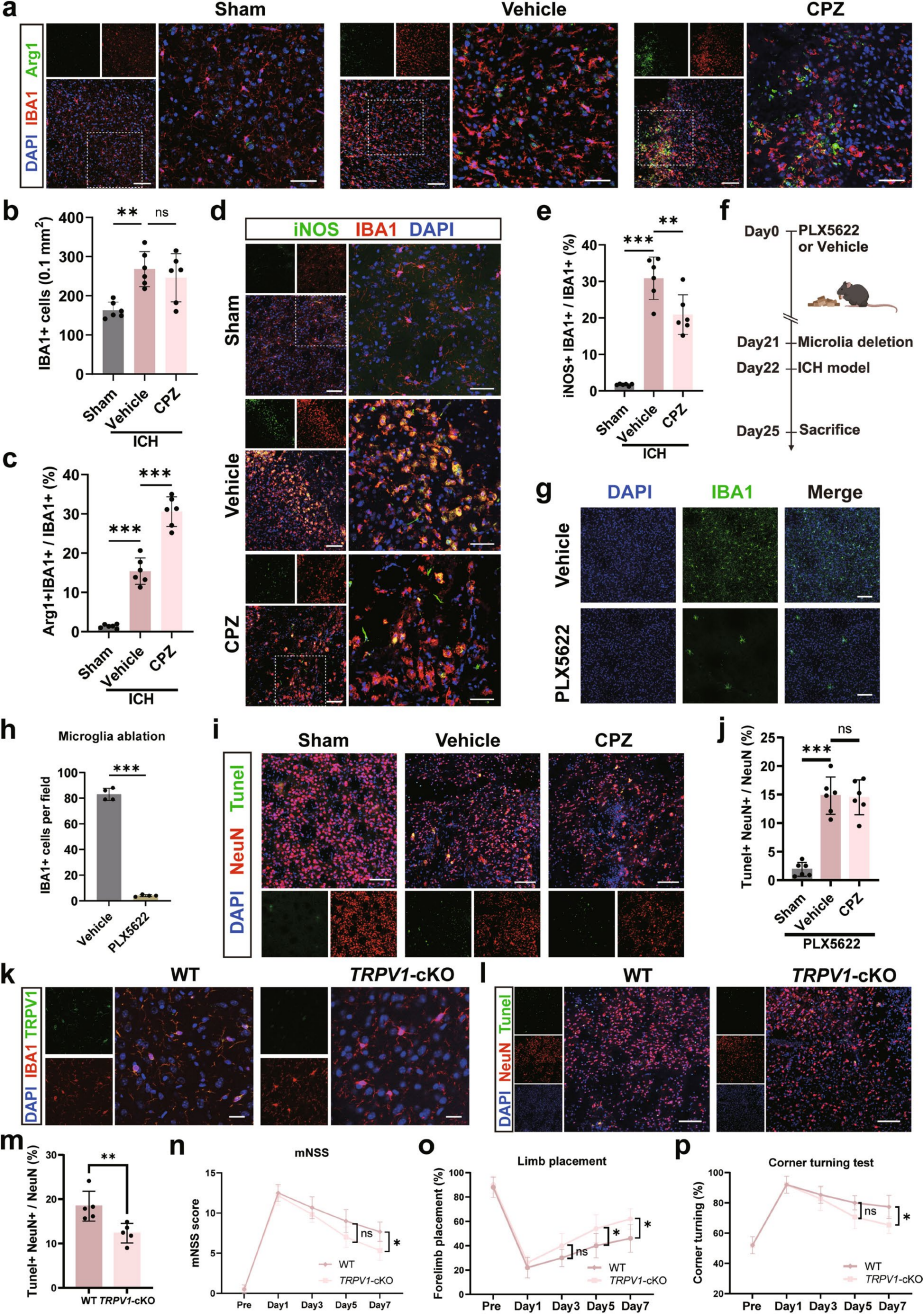
The function of TRPV1 in ICH was microglia‐dependent. (a) Images showing Arg1 costained with IBA1 in the perihematomal area 3 days post‐ICH. (b) Quantification of the number of microglia (IBA1+) cells. (c) Statistical analysis of the proportion of Arg + IBA1+ regulatory microglia. (d) Images of iNOS costained with IBA1 3 days after ICH. Scale bar is 100 μm in merged images and 50 μm in ZOOM images in (a) and (d). (e) Statistical analysis of the proportion of iNOS+IBA1+ proinflammatory microglia. (f) Schematic diagram of microglial deletion. Created with BioRender.com. (g) Images of IBA1(green) staining before and after PLX5622 feeding. Scale bar is 100 μm. (h) Quantitative analysis of the number of IBA1‐positive cells in (b). (i) Images showing co‐staining of TUNEL (green) with neurons (NeuN, red) 3 days after ICH under different conditions following microglia depletion. Scale bar is 100 μm. (j) Quantitative analysis of apoptotic neurons (TUNEL+ NeuN+) in (d). (k) Representative images of co‐staining of TRPV1 with IBA1 in TRPV1 microglia‐specific knockout mice (TRPV1‐cKO, TRPV1^*flox/flox*^; Cx3cr1^*Cre*^) and control wild‐type (WT, TRPV1^*flox/flox*^) mice. The scale bar is 20 μm. (l) Representative images of TUNEL (green) and NeuN (red) colocalization in neurons within the perihematomal area, 3 days post‐ICH in TRPV1‐cKO and WT mice. The scale bar is 100 μm. (m) Quantitative analysis of apoptotic neurons (TUNEL+ NeuN+) in (g). (n–p) Modified neurological deficit score (mNSS), forelimb placement (FP) test and corner turning test 1, 3, 5 and 7 days after ICH. Data were expressed as mean ± SD. *p < 0.05, **p < 0.01, ***p < 0.001 vs. vehicle group. *n* = 6/group in (a, d and i), *n* = 5/group in (l–p), *n* = 4/group in (g).

Since microglia are key responders in ICH [35] and predominantly upregulate TRPV1, we investigated whether TRPV1 blockade protects neurons by modulating their function. CPZ treatment did not change the total number of IBA1^+^ microglia at Day 3 post‐ICH (*p* > 0.05, Figure 4a,b) but significantly promoted their phenotypic shift: it increased the proportion of IBA1^+^Arg1^+^ regulatory microglia (*p* < 0.05, Figure 4a,c) while reducing IBA1^+^iNOS^+^ pro‐inflammatory microglia (*p* < 0.05, Figure 4d,e). These results indicate that CPZ reprograms microglia from a pro‐inflammatory to a regulatory state.

To establish the microglial dependency of CPZ's effects, we depleted microglia using the CSF1R inhibitor PLX5622 (Figure 4f), which effectively reduced the microglial population (*p* < 0.05, Figure 4g,h). Depletion exacerbated ICH‐induced neuronal apoptosis (*p* < 0.05, Figure 4i,j) and abolished the neuroprotective effect of CPZ, as apoptosis levels showed no difference between CPZ‐ and vehicle‐treated groups (*p* > 0.05, Figure 4i,j). These results demonstrate that microglia are essential for CPZ's anti‐apoptotic effect, indicating microglia‐dependent mediation.

To definitively establish microglial TRPV1 as the key mediator in ICH, we generated a microglia‐specific TRPV1 cKO model. Through genotyping and immunohistochemical staining, we validated effective TRPV1 knockout in microglia (Figure 4k). TRPV1 cKO mice exhibited reduced neuronal apoptosis (*p* < 0.05, Figure 4l,m) and improved neurological function across multiple behavioral tests on Day 7 post‐ICH (*p* < 0.05, Figure 4n–p). This neuroprotective phenotype mirrored the effects of CPZ treatment, confirming that TRPV1 exacerbates ICH injury primarily through its actions in microglia.

To further confirm that CPZ acts specifically through microglial TRPV1, we treated microglia‐specific TRPV1 cKO mice with CPZ after ICH. While a subset of neurons retained TRPV1 expression (Figure S4a), CPZ failed to confer additional protection beyond microglial TRPV1 deletion alone: neuronal apoptosis (Figure S4b,c) and mitophagy markers (LC3B‐VDAC and Parkin‐VDAC colocalization, Figure S4d–g) were comparable between vehicle‐ and CPZ‐treated cKO groups (*p* > 0.05). These results provide direct genetic evidence that microglial TRPV1 is indispensable for CPZ's protective effects.

We also performed in vitro validation using a transwell system to determine CPZ's microglia‐specificity (Figure S5a). Conditioned medium from Hb‐stimulated BV2 microglia reduced mitochondrial membrane potential in HT22 neurons (JC‐1 staining—an early marker of apoptosis, Figure S5b,c, *p* < 0.05) and increased neuronal apoptosis (cleaved caspase‐3, Figure S5d,e, *p* < 0.05). These effects were significantly attenuated when BV2 cells were pretreated with CPZ, confirming indirect neuroprotection through microglial modulation. CPZ pretreatment also reduced IL‐1β and TNF‐α levels in BV2‐conditioned medium (Figure S5f,g, *p* < 0.05).

Given that mitochondrial damage can lead to the release of mitochondrial DNA (mtDNA), which acts as a damage‐associated molecular pattern (DAMP) to amplify inflammation [36, 37, 38], we next examined cytosolic mtDNA levels. As shown in Figure S5h, Hb stimulation triggered mtDNA release into the cytoplasm of BV2 microglia (Picogreen in cytoplasm), suggesting that mtDNA leakage may contribute to the pro‐inflammatory microglial phenotype. However, no significant changes in the number of mitophagosomes (*p* > 0.05, Figure S5i,j) or in the colocalization of Parkin with MitoTracker (*p* > 0.05, Figure S5k,l) were observed in HT22 neurons. These findings indicate that CPZ protects neurons indirectly by suppressing microglial mtDNA release and pro‐inflammatory cytokines rather than directly modulating neuronal autophagy, consistent with our in vivo data showing CPZ shifts microglia toward a regulatory phenotype (Figure 4a–e).

### 3.6 Blockade of TRPV1 Alleviated Excessively Elevated Mitophagy in ICH

**FIGURE 5:**
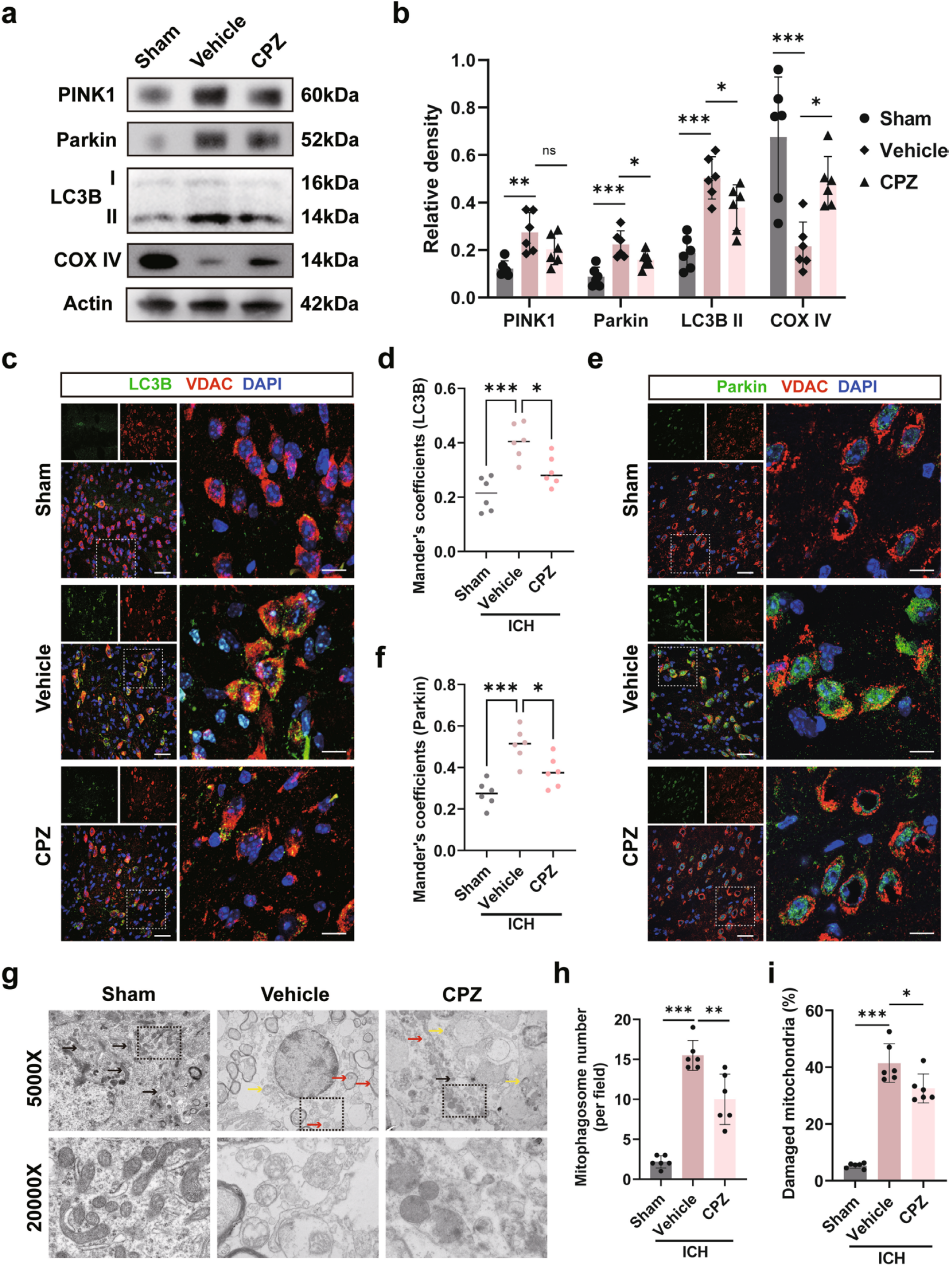
Blockade of TRPV1 alleviated excessive elevation of mitophagy in ICH. (a) Western blot images of autophagy markers LC3B II, PINK1, Parkin and mitochondrial marker COX IV. (b) Quantitative analysis of LC3B II, PINK1, Parkin and COX IV 3 days post‐ICH. (c and d) Images of autophagic vesicles (LC3B, green) costained with mitochondria (VDAC, red) and quantitative analysis of Mander's coefficient for LC3B with VDAC 3 days post‐ICH. (e and f) Images of the mitophagy marker (Parkin, green) costained with mitochondria (VDAC, red) and quantitative analysis of Mander's coefficient for Parkin with VDAC 3 days post‐ICH. Scale bar is 30 μm in (c) and (e), scale bar is 10 μm in ZOOM images of (c) and (e). (g) Images of TEM of the perihematomal tissue 3 days post‐ICH, with the top row at ×5000 magnification and the bottom row at ×20,000 magnification. The black arrows represent normal mitochondria, the yellow arrows represent damaged mitochondria, and the red arrows represent mitophagosomes. (h and i) Quantitative analysis of the number of mitophagosomes and the ratio of damaged mitochondria. *p < 0.05, **p < 0.01, ***p < 0.001 vs. vehicle, *n* = 6/group.

We next investigated the downstream mechanisms of microglial TRPV1 activation. TRPV1 is closely associated with autophagy [16, 39], an intracellular phagocytic activity. Although autophagy is significantly elevated after ICH, its specific role and underlying mechanisms remain unclear [40]. Western blot analysis showed that ICH significantly upregulated the autophagy‐related proteins Parkin, PINK1, and LC3B‐II (*p* < 0.05, Figure 5a,b), indicating enhanced autophagy. CPZ treatment reduced the levels of Parkin and LC3B‐II (*p* < 0.05, Figure 5a,b), suggesting suppressed autophagic activity. Furthermore, CPZ partially reversed the ICH‐induced decrease in the mitochondrial marker COX IV (*p* < 0.05, Figure 5a,b), suggesting that TRPV1 activation contributes to mitochondrial loss via excessive autophagy.

Given the critical role of mitochondrial function in microglial phagocytosis during ICH [14], we specifically investigated mitophagy. Immunofluorescence analysis revealed that ICH significantly enhanced the colocalization of LC3B with VDAC (*p* < 0.05, Figure 5c,d), indicating enhanced mitophagic flux. This increase was significantly attenuated by CPZ treatment (*p* < 0.05, Figure 5c,d). Similarly, CPZ also reduced the ICH‐induced colocalization (Manders coefficient) between the mitophagy receptor Parkin and VDAC (*p* < 0.05, Figure 5e,f). These data demonstrate that TRPV1 blockade suppresses ICH‐induced mitophagy activation. Direct visualization by TEM confirmed these findings: vehicle‐treated group exhibited a substantial increase in mitophagosomes (*p* < 0.05, Figure 5g–i, red arrows) and damaged mitochondria (yellow arrows), which was significantly reduced by CPZ treatment.

### 3.7 CPZ Reduced Mitophagy and Maintained Mitochondrial Integrity in Microglia

**FIGURE 6:**
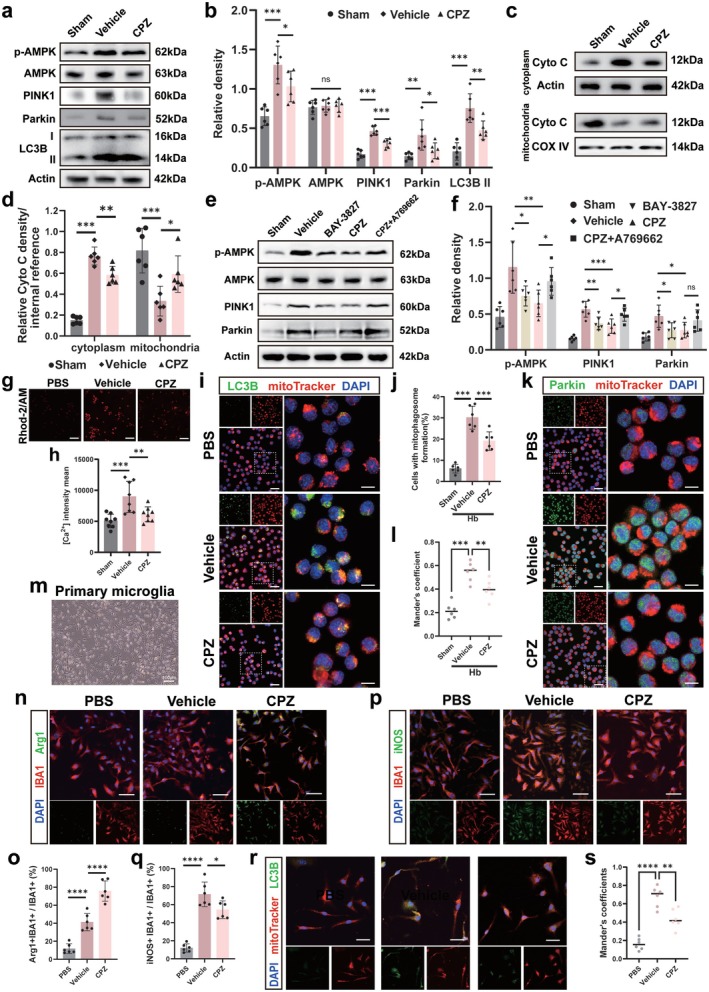
CPZ reduced mitophagy and maintained mitochondrial integrity in microglial cells. (a) Western blot images for AMPK phosphorylation and mitophagy markers in BV2 cells. (b) Relative expression levels of p‐AMPK, AMPK, PINK1, Parkin and LC3B II after 24 h of Hb treatment. (c) Western blot images for the mitochondrial content marker Cyto C. (d) Relative amounts of Cyto C in mitochondria or cytoplasm under different interventions. (e) Western blot images for AMPK phosphorylation and mitophagy markers in BV2 cells treated with AMPK inhibitors BAY3827 or agonist A769662 (with CPZ). (f) Relative expression levels of p‐AMPK, PINK1 and Parkin after 24 h of Hb treatment in different groups. (g) Representative image of Rhod‐2/AM staining of BV2 cells in different groups. (h) Quantitative analysis of average calcium signal intensity in BV2 cells. Scale bar = 100 μm. (i and j) Images of autophagic vesicles (LC3B, green) costained with mitochondria (mitoTracker, red) in BV2 cells and quantitative analysis of mitochondrial autophagic vesicles. (k and l) Images of mitophagy marker Parkin (green) costained with mitochondria (mitoTracker, red) and quantitative analysis of Mander's coefficient for Parkin with mitoTracker. Scale bar is 30 μm in (i) and (k), scale bar is 10 μm in ZOOM images of (i) and (k). (m) Representative bright‐field image of primary microglia, showing microglia with multiple processes. Scale bar is 100 μm. (n and o) Representative images of Arg1‐positive cells of microglia under different treatment conditions and quantitative analysis of the proportion of Arg1‐positive cells. Scale bar is 50 μm. (p and q) Representative images of iNOS‐positive cells of microglia under different treatment conditions and quantitative analysis of the proportion of iNOS‐positive cells. Scale bar is 50 μm. (r and s) Images of autophagic vesicles (LC3B, green) costained with mitochondria (mitoTracker, red) and quantitative analysis of Mander's coefficient for LC3B with mitoTracker. Scale bar is 30 μm. *p < 0.05, **p < 0.01, ***p < 0.001, ****p < 0.0001 vs. vehicle, *n* = 6/group. ^#^ *p* < 0.05 CPZ vs. CPZ + A769662, *n* = 6/group in (a–f) and (i–s), *n* = 8/group in (g).

We next validated the role of TRPV1 in ICH‐associated autophagy using an in vitro model. Hb treatment significantly upregulated LC3B, PINK1, and Parkin expression (*p* < 0.05, Figure 6a,b), recapitulating the in vivo findings, and these increases were suppressed by CPZ. Furthermore, cellular fractionation revealed that Hb‐induced cytochrome C (Cyto C) release from mitochondria into the cytoplasm was also mitigated by CPZ (*p* < 0.05, Figure 6c,d). These findings confirm that CPZ stabilizes mitochondrial structure by inhibiting TRPV1‐driven autophagy in microglia.

TRPV channel activation is known to trigger AMPK signaling [17], a key regulator of autophagy that responds to calcium flux [41]. Accordingly, Hb stimulation increased AMPK phosphorylation, an effect attenuated by CPZ (*p* < 0.05, Figure 6a,b). Pharmacological inhibition of AMPK (BAY‐3827) downregulated PINK1 and Parkin, similar to CPZ, while AMPK activation (A769662) partially reversed CPZ's effects (*p* < 0.05, Figure 6e,f). Since TRPV1 activation elevates intracellular Ca^2+^, we measured calcium levels and confirmed that CPZ reduced the Hb‐induced Ca^2+^ influx (*p* < 0.05; Figure 6g,h). Together, these results demonstrate that TRPV1 activation promotes autophagy primarily via the Ca^2+^‐AMPK‐PINK1 pathway.

We also analyzed the cellular localization of LC3B and Parkin. Hb stimulation significantly increased the colocalization of LC3B with VDAC, which was attenuated by CPZ treatment (*p* < 0.05, Figure 6i,j). Similarly, the colocalization index of Parkin with VDAC demonstrated the same pattern (*p* < 0.05, Figure 6k,l), confirming that TRPV1 blockade suppresses Hb‐induced mitophagy.

To determine whether CPZ selectively inhibits mitophagy or broadly suppresses autophagy, we performed subcellular fractionation to analyze autophagic activity in mitochondrial versus cytosolic compartments. Following Hb stimulation, CPZ significantly reduced PINK1 and Parkin levels in both mitochondrial and cytosolic fractions (Figure S6a–d, *p* < 0.05), indicating that CPZ suppresses global autophagy flux rather than selectively targeting mitophagy.

Despite this autophagy suppression, we next asked whether phagocytic function—critical for hematoma clearance—was compromised. Using PKH26‐labeled red blood cell phagocytosis assays, we found that CPZ‐treated BV2 cells exhibited significantly enhanced uptake compared to vehicle‐treated cells, as evidenced by the increased percentage of phagocytic cells and higher number of engulfed RBCs per cell (Figure S6e–g, *p* < 0.05). These results demonstrate that while CPZ suppresses global autophagy, it paradoxically preserves—and even enhances—phagocytic capacity, likely by preventing the mitochondrial dysfunction caused by excessive autophagy (Figures 5 and 6).

### 3.8 Excessive Autophagy in Microglia Induces Neural Injury in ICH

We validated the concordant responses of primary microglia and BV2 cells in the ICH model. Primary microglia, isolated by gradient centrifugation [42] and identified by morphology and IBA1 staining (Figure 6m,n,p), exhibited a CPZ‐induced protective phenotype. This was evidenced by an increase in Arg1^+^ cells and a decrease in iNOS^+^ cells (*p* < 0.05, Figure 6n–q), consistent with in vivo data (Figure 4a,d). CPZ also inhibited autophagosome formation (*p* < 0.05, Figure 6r–s), further corroborating our findings in a primary system.

To further corroborate our findings, we knocked down TRPV1 expression in BV2 cells via siRNAs (*p* < 0.05, Figure S6h,i). TRPV1 knockdown effectively reduced the number of Hb‐induced mitophagosomes (*p* < 0.05, Figure S6j,k), reinforcing that TRPV1 blockade suppresses aberrant autophagy following ICH.

## 4 Discussion

TRPV1 expression is not restricted to peripheral sensory neurons but is also detected in various brain regions, including the cortex, hypothalamus, cerebellum, and striatum [43, 44, 45]. In our experiments, TRPV1 was substantially increased after ICH and remained elevated for 1 week. Immunohistochemistry identified microglia as the primary TRPV1‐positive cells post‐ICH, with limited neuronal expression and sparse detection in astrocytes or oligodendrocytes. This pattern differs from the resting state, where TRPV1 is predominantly neuronal [46, 47].

The role of TRPV1 varies across neurological disorders. In Parkinson's disease, TRPV1 activation suppresses excessive glial activation and protects dopaminergic neurons [27, 28]. In Alzheimer's disease, microglial TRPV1 activation mitigates mitochondrial damage [48]. The role of TRPV1 in ischemic events is complicated. Systemic or intracerebroventricular activation can reduce cortical and thalamic damage, potentially through hypothermia [49, 50], yet TRPV1 knockout mice exhibit smaller infarcts and fewer neurological deficits [51]. Notably, TRPV1 blockade consistently improves outcomes in hemorrhagic models [52, 53], aligning with our ICH results.

Our finding that TRPV1 activation exacerbates ICH injury appears to contrast with neuroprotective effects reported in Parkinson's disease [28]. This discrepancy likely reflects disease context: in chronic neurodegeneration, moderate TRPV1 activation may promote protective autophagy and metabolite clearance [27], whereas in acute ICH, rapid and excessive TRPV1 activation drives pathologically elevated mitophagy that exceeds homeostatic needs.

A recent study conducted by Chen et al. demonstrated that TRPV1 inhibition or deletion attenuated brain damage, neurodegeneration, and microglial activation following ICH [11]. They found that TRPV1 inhibition reduced calcium influx and suppressed the phosphorylation of CaMKII in cultured neurons. Chen et al. demonstrated that pre‐ICH CAP administration improved outcomes; we note this is not contradictory—pre‐ICH CAP likely induces TRPV1 desensitization [26], achieving functional inhibition—consistent with our conclusion that TRPV1 inhibition is protective in ICH. Moreover, we employed microglia‐specific conditional knockout and in vitro knockdown models to establish that microglial TRPV1 inhibition alone is sufficient to improve ICH outcomes. Together, these reports provide a mechanistic link between microglial TRPV1 blockade and functional recovery.

CPZ significantly attenuated BBB disruption after ICH, evidenced by upregulated ZO‐1 and claudin‐5 (Figure S3). This protective effect is likely mediated through suppression of microglial neuroinflammation, given that TRPV1 expression after ICH is predominantly microglial with minimal endothelial colocalization (Figure 1c,d). CPZ shifted microglia toward a regulatory phenotype (Figure 4a–e), reducing inflammatory cytokine release and mitigating tight junction disruption [54]. However, direct endothelial effects cannot be excluded, as TRPV1 is expressed in brain endothelial cells and TRPV1 inhibition can directly preserve tight junction proteins and reduce endothelial apoptosis in other brain injury models [55, 56]. Endothelial‐specific investigations represent an important future direction to delineate whether direct endothelial TRPV1 signaling contributes to BBB protection following ICH.

TRPV1 receptors modulate brain learning and memory functions through regulating neurotransmitter release and triggering long‐term synaptic depression [57, 58]. In this study, CPZ‐treated ICH mice performed better in mNSS, foot fault, and corner turn tests, possibly due to enhanced neuronal survival and modulated electrophysiology. TRPV1 activity is also involved in neurogenesis and neural stem cell differentiation [59]. Although we did not assess long‐term neurogenesis, Morris water maze results indicated improved learning and memory in CPZ‐treated ICH mice, suggesting that TRPV1 activation may impair neurogenesis after ICH and that its inhibition could be beneficial.

The essential role of microglial TRPV1 was further confirmed by our cKO rescue experiments. While a subset of neurons retained TRPV1 expression, CPZ treatment failed to reduce neuronal apoptosis or mitophagy in microglia‐specific TRPV1 cKO mice, demonstrating that microglial TRPV1 is necessary for CPZ's therapeutic effects. This aligns with our transwell experiments showing that CPZ‐conditioned medium from microglia protected neurons by reducing inflammatory cytokines (IL‐1β, TNF‐α) and mtDNA release (Figure S5), rather than directly modulating neuronal autophagy.

Autophagy plays context‐dependent roles in neurological diseases [60, 61]. TRPV1 activation induces protective autophagy in vascular smooth muscle cells to reduce lipid deposition [62], yet in tumors, it drives autophagy‐mediated chemoresistance [39], and in acute toxicity, exacerbates injury [16]. This duality is evident in microglial autophagy—it can promote inflammation in demyelinating models [63] or facilitate debris clearance in multiple sclerosis [64]. The observation that TRPV1 inhibition suppresses mitophagy while enhancing hematoma clearance may appear paradoxical given shared machinery with LAP, but emerging evidence supports a nonlinear “U‐shaped” relationship [24, 25]. ICH triggers excessive mitophagy that impairs microglial function; by attenuating this hyper‐mitophagy, CPZ preserves mitochondrial integrity and maintains metabolic fitness for phagocytosis, supported by our in vitro phagocytosis data (Figure S6).

Subcellular fractionation revealed that CPZ suppresses global autophagy flux rather than selectively inhibiting mitophagy. Combined with enhanced phagocytic function, this supports an energy‐centric interpretation: excessive mitophagy depletes mitochondria, causing bioenergetic failure [24, 25]. By attenuating overactivated autophagy, CPZ preserves mitochondrial integrity and ATP supply—consistent with the U‐shaped model.

Recent studies show that mtDNA from damaged mitochondria activates microglial inflammation via cGAS‐STING and AIM2 inflammasome pathways [36, 37, 38, 65]. Our finding that Hb increases cytosolic mtDNA in microglia (Figure S5h) suggests mtDNA release amplifies pro‐inflammatory responses. By preserving mitochondrial integrity, CPZ may reduce mtDNA leakage, thereby suppressing this pro‐inflammatory amplification loop—a hypothesis that warrants further investigation.

We also identified AMPK as a key mediator of TRPV1‐driven autophagy. TRPV1 activation raised intracellular Ca^2+^ and subsequent AMPK phosphorylation [17]. Consistent with earlier reports [16], TRPV1 opening increased p‐AMPK levels in BV2 cells, an effect blocked by CPZ or AMPK inhibition. Furthermore, the AMPK agonist partly reversed CPZ's suppression of autophagy, supporting a TRPV1–Ca^2+^–AMPK–PINK1–Parkin mitophagy pathway in microglia.

While our data demonstrate TRPV1 upregulation after ICH, whether this reflects enhanced channel function remains an important question. TRPV1 activity is dynamically regulated: prolonged activation can induce desensitization via calcineurin [66, 67], while phosphorylation by PKA/PKC/CaMKII enhances responsiveness [68, 69]. Several observations suggest TRPV1 function is likely augmented in ICH: (1) elevated intracellular Ca^2+^ after Hb stimulation (Figure 6g,h) activates Ca^2+^‐dependent kinases that sensitize TRPV1 and (2) the inflammatory ICH milieu contains mediators that promote TRPV1 sensitization [70]. Future studies directly assessing TRPV1 phosphorylation and electrophysiological properties would further clarify its functional status in ICH.

Several limitations of this study should be noted. First, while TRPV1 is reported in endothelial cells [71], we observed minimal peri‐hematoma endothelial expression, a finding that requires validation in other ICH models beyond the collagenase method. Meanwhile, the scope of TRPV1‐modulated microglial autophagy and its impact on microglia–neuron communication are not fully delineated and need further validation, including direct demonstration of neuronal phenotype alterations. Additionally, genetic model data are preliminary. Future studies should employ more detailed strategies in conditional knockout mice to fully elucidate the role of microglial TRPV1 in ICH.

## 5 Conclusion

In summary, our study demonstrates that TRPV1 upregulation following ICH exacerbates brain injury. Blocking TRPV1 with CPZ shifted microglia toward a regulatory phenotype, promoted neuronal survival, and improved functional recovery. This protection was mediated by the suppression of excessive microglial mitophagy via the AMPK‐PINK1‐Parkin pathway. Therefore, targeting TRPV1‐driven autophagy represents a promising therapeutic strategy for acute ICH management.

## Author Contributions

Conceptualization: Kezhu Chen and Jun Zeng. Methodology: Kezhu Chen, Xiangyang Deng, Baoye Sun, Junjie Zhong, Peng Wang, Fengshi Li and Junwei Ren. Validation: Junjie Zhong, Pengjie Hong, Peng Wang, Fengshi Li, Qisheng Tang, Tongming Zhu and Jianhong Zhu. Formal analysis: Kezhu Chen, Jun Zeng, Jingyu Yu, Tianwen Li, Peng Wang and Quan Zhang. Investigation: Kezhu Chen, Xiangyang Deng, Baoye Sun, Jingyu Yu, Tianwen Li, and Quan Zhang. Resources: Baoye Sun, Jingyu Yu, Tianwen Li, Junjie Zhong, Pengjie Hong, Fengshi Li and Junwei Ren. Data curation: Kezhu Chen, Xiangyang Deng and Jun Zeng. Writing – original draft: Kezhu Chen and Jun Zeng. Writing – review and editing: Pengjie Hong, Qisheng Tang, Tongming Zhu and Jun Zeng. Visualization: Kezhu Chen and Xiangyang Deng. Supervision: Jun Zeng. Project administration: Qisheng Tang, Tongming Zhu and Jianhong Zhu. Funding acquisition: Jun Zeng. All authors read and approved the final version of the manuscript.

## Funding

This work was supported by grants from National Natural Science Foundation and Ministry of Science and Technology of China (82571833, 92168103, 32171417, 82571577, 2018YFA0107900) and the Shanghai Municipal Government, the Peak Disciplines (Type IV) of Institutions of Higher Learning in Shanghai (2019CXJQ01), and the Natural Science Foundation of Shanghai (25ZR1401044).

## Ethics Statement

All procedures were approved by the Animal Care Committee of Laboratory Animal Center Fudan University (Approval ID: 202509048Z) and followed ARRIVE guidelines.

## Conflicts of Interest

The authors declare no conflicts of interest.

## Supporting information


**Table S1:** Antibodies used for western blotting.

**Table S2:** Antibodies used for immunostaining.

**Table S3:** siRNA sequences for TRPV1.

**Figure S1:** Schematic diagram of animal experiment design.
**Figure S2:** The effects of different doses of CAP and CPZ on mNSS scores in ICH mice.
**Figure S3:** The effects of CPZ on BBB in ICH mice.
**Figure S4:** CPZ exerts neuroprotective effects through microglial TRPV1 after ICH.
**Figure S5:** BV2 conditioned medium was associated with HT22 survival.
**Figure S6:** TRPV1 regulated mitophagy and endocytosis in BV2 cells.

## Data Availability

The data that support the findings of this study are available from the corresponding author upon reasonable request.

## References

[1] B. A. Gross , B. T. Jankowitz , and R. M. Friedlander , “Cerebral Intraparenchymal Hemorrhage: A Review,” JAMA 321, no. 13 (2019): 1295–1303, 10.1001/jama.2019.2413. 30938800

[2] M. Zille , T. D. Farr , R. F. Keep , C. Römer , G. Xi , and J. Boltze , “Novel Targets, Treatments, and Advanced Models for Intracerebral Haemorrhage,” eBioMedicine 76 (2022): 103880, 10.1016/j.ebiom.2022.103880. 35158309 PMC8850756

[3] L. Wang , L. Zhang , K. Wang , et al., “Microglial Lcn2 Knockout Enhances Chronic Intracerebral Hemorrhage Recovery by Restoring Myelin and Reducing Inflammation,” Theranostics 15, no. 10 (2025): 4763–4784, 10.7150/thno.109440. 40225581 PMC11984404

[4] J. A. Kauer and H. E. Gibson , “Hot Flash: TRPV Channels in the Brain,” Trends in Neurosciences 32, no. 4 (2009): 215–224, 10.1016/j.tins.2008.12.006. 19285736

[5] J. A. Cohen , T. N. Edwards , A. W. Liu , et al., “Cutaneous TRPV1(+) Neurons Trigger Protective Innate Type 17 Anticipatory Immunity,” Cell 178, no. 4 (2019): 919–932.e14, 10.1016/j.cell.2019.06.022. 31353219 PMC6788801

[6] M. Balood , M. Ahmadi , T. Eichwald , et al., “Nociceptor Neurons Affect Cancer Immunosurveillance,” Nature 611, no. 7935 (2022): 405–412, 10.1038/s41586-022-05374-w. 36323780 PMC9646485

[7] M. C. Marrone , A. Morabito , M. Giustizieri , et al., “TRPV1 Channels Are Critical Brain Inflammation Detectors and Neuropathic Pain Biomarkers in Mice,” Nature Communications 8 (2017): 15292, 10.1038/ncomms15292. PMC5436240 28489079

[8] Y. Zhang , B. Hou , P. Liang , et al., “TRPV1 Channel Mediates NLRP3 Inflammasome‐Dependent Neuroinflammation in Microglia,” Cell Death & Disease 12, no. 12 (2021): 1159, 10.1038/s41419-021-04450-9. 34907173 PMC8671551

[9] Y. Awad‐Igbaria , A. Ben‐Menashe , R. Sakas , et al., “Novel Insight Into TRPV1‐Induced Mitochondrial Dysfunction in Neuropathic Pain,” Brain: A Journal of Neurology 148, no. 7 (2025): 2563–2578, 10.1093/brain/awaf044. 39901826

[10] T. Schilling and C. Eder , “Importance of the Non‐Selective Cation Channel TRPV1 for Microglial Reactive Oxygen Species Generation,” Journal of Neuroimmunology 216, no. 1–2 (2009): 118–121, 10.1016/j.jneuroim.2009.07.008. 19683814

[11] C. C. Chen , C. H. Ke , C. H. Wu , et al., “Transient Receptor Potential Vanilloid 1 Inhibition Reduces Brain Damage by Suppressing Neuronal Apoptosis After Intracerebral Hemorrhage,” Brain Pathology 34, no. 5 (2024): e13244, 10.1111/bpa.13244. 38308041 PMC11328348

[12] Q. Bai , M. Xue , and V. W. Yong , “Microglia and Macrophage Phenotypes in Intracerebral Haemorrhage Injury: Therapeutic Opportunities,” Brain: A Journal of Neurology 143, no. 5 (2020): 1297–1314, 10.1093/brain/awz393. 31919518

[13] Y. Li , H. Zhou , X. He , et al., “Impaired Microglial Glycolysis Promotes Inflammatory Responses After Intracerebral Haemorrhage via HK2‐Dependent Mitochondrial Dysfunction,” Journal of Advanced Research 73 (2025): 575–591, 10.1016/j.jare.2024.08.016. 39142439 PMC12225926

[14] X. Yan , M. He , H. Huang , et al., “Endogenous H(2)S Targets Mitochondria to Promote Continual Phagocytosis of Erythrocytes by Microglia After Intracerebral Hemorrhage,” Redox Biology 56 (2022): 102442, 10.1016/j.redox.2022.102442. 35998432 PMC9420393

[15] T. Huang , Y. Lin , Q. Pang , W. Shen , X. Chen , and F. Tu , “The Synergistic Effect of TRPV1 on Oxidative Stress‐Induced Autophagy and Apoptosis in Microglia,” Analytical Cellular Pathology (Amsterdam) 2021 (2021): 7955791, 10.1155/2021/7955791. 34336554 PMC8298174

[16] M. Chen , X. Dong , H. Deng , et al., “Targeting TRPV1‐Mediated Autophagy Attenuates Nitrogen Mustard‐Induced Dermal Toxicity,” Signal Transduction and Targeted Therapy 6, no. 1 (2021): 29, 10.1038/s41392-020-00389-z. 33487631 PMC7829253

[17] M. Li , C. S. Zhang , Y. Zong , et al., “Transient Receptor Potential V Channels Are Essential for Glucose Sensing by Aldolase and AMPK,” Cell Metabolism 30, no. 3 (2019): 508–524.e12, 10.1016/j.cmet.2019.05.018. 31204282 PMC6720459

[18] Z. Fan , L. Du , L. Yin , et al., “Mitochondrial TRPV1 Exacerbates Cognitive Deficits in Sepsis‐Associated Encephalopathy by Driving Microglial Metabolic Reprogramming,” Free Radical Biology & Medicine 247 (2026): 448–468, 10.1016/j.freeradbiomed.2026.02.033. 41692320

[19] H. Shi , J. Wang , J. Wang , Z. Huang , and Z. Yang , “IL‐17A Induces Autophagy and Promotes Microglial Neuroinflammation Through ATG5 and ATG7 in Intracerebral Hemorrhage,” Journal of Neuroimmunology 323 (2018): 143–151, 10.1016/j.jneuroim.2017.07.015. 28778418

[20] A. Xu , Y. Liu , B. Wang , et al., “Ceramide Synthase 6 Induces Mitochondrial Dysfunction and Apoptosis in Hemin‐Treated Neurons by Impairing Mitophagy Through Interacting With Sequestosome 1,” Free Radical Biology & Medicine 227 (Feb 1 2025): 282–295, 10.1016/j.freeradbiomed.2024.12.018. 39643132

[21] M. S. D'Arcy , “Cell Death: A Review of the Major Forms of Apoptosis, Necrosis and Autophagy,” Cell Biology International 43, no. 6 (2019): 582–592, 10.1002/cbin.11137. 30958602

[22] J. Lu , C. Wang , X. Cheng , et al., “A Breakdown in Microglial Metabolic Reprogramming Causes Internalization Dysfunction of α‐Synuclein in a Mouse Model of Parkinson's Disease,” Journal of Neuroinflammation 19, no. 1 (2022): 113, 10.1186/s12974-022-02484-0. 35599331 PMC9124408

[23] I. Choi , M. Wang , S. Yoo , et al., “Autophagy Enables Microglia to Engage Amyloid Plaques and Prevents Microglial Senescence,” Nature Cell Biology 25, no. 7 (2023): 963–974, 10.1038/s41556-023-01158-0. 37231161 PMC10950302

[24] J. Wu , J. Liu , Y. Li , et al., “Microglial Autophagy and Mitophagy in Ischemic Stroke: From Dual Roles to Therapeutic Modulation,” Biology‐Basel 14, no. 9 (2025): 1269, 10.3390/biology14091269. 41007413 PMC12467092

[25] Y. Liu , M. Wang , X. O. Hou , and L. F. Hu , “Roles of Microglial Mitophagy in Neurological Disorders,” Frontiers in Aging Neuroscience 14 (2022): 979869, 10.3389/fnagi.2022.979869. 36034136 PMC9399802

[26] S. X. Shi , Y. J. Li , K. Shi , K. Wood , A. F. Ducruet , and Q. Liu , “IL (Interleukin)‐15 Bridges Astrocyte‐Microglia Crosstalk and Exacerbates Brain Injury Following Intracerebral Hemorrhage,” Stroke 51, no. 3 (2020): 967–974, 10.1161/strokeaha.119.028638. 32019481

[27] E. Bok , Y. C. Chung , K. S. Kim , H. H. Baik , W. H. Shin , and B. K. Jin , “Modulation of M1/M2 Polarization by Capsaicin Contributes to the Survival of Dopaminergic Neurons in the Lipopolysaccharide‐Lesioned Substantia Nigra In Vivo,” Experimental & Molecular Medicine 50, no. 7 (2018): 1–14, 10.1038/s12276-018-0111-4. PMC6030094 29968707

[28] Y. C. Chung , J. Y. Baek , S. R. Kim , et al., “Capsaicin Prevents Degeneration of Dopamine Neurons by Inhibiting Glial Activation and Oxidative Stress in the MPTP Model of Parkinson's Disease,” Experimental & Molecular Medicine 49, no. 3 (2017): e298, 10.1038/emm.2016.159. 28255166 PMC5382554

[29] X. Deng , J. Ren , K. Chen , et al., “Mas Receptor Activation Facilitates Innate Hematoma Resolution and Neurological Recovery After Hemorrhagic Stroke in Mice,” Journal of Neuroinflammation 21, no. 1 (2024): 106, 10.1186/s12974-024-03105-8. 38658922 PMC11041011

[30] P. Jin , S. Deng , P. Sherchan , et al., “Neurokinin Receptor 1 (NK1R) Antagonist Aprepitant Enhances Hematoma Clearance by Regulating Microglial Polarization via PKC/p38MAPK/NFκB Pathway After Experimental Intracerebral Hemorrhage in Mice,” Neurotherapeutics 18, no. 3 (2021): 1922–1938, 10.1007/s13311-021-01077-8. 34244927 PMC8608951

[31] M. Hanifa , M. Suri , H. Singh , R. Gagnani , A. S. Jaggi , and A. Bali , “Dual Role of TRPV1 Channels in Cerebral Stroke: An Exploration From a Mechanistic and Therapeutic Perspective,” Molecular Neurobiology 61 (2024): 10574–10592, 10.1007/s12035-024-04221-5. 38760620

[32] T. K. E. Maximiano , J. A. Carneiro , V. Fattori , and W. A. Verri , “TRPV1: Receptor Structure, Activation, Modulation and Role in Neuro‐Immune Interactions and Pain,” Cell Calcium 119 (2024): 102870, 10.1016/j.ceca.2024.102870. 38531262

[33] M. Wu , G. Song , J. Li , et al., “Innervation of Nociceptor Neurons in the Spleen Promotes Germinal Center Responses and Humoral Immunity,” Cell 187, no. 12 (2024): 2935–2951.e19, 10.1016/j.cell.2024.04.027. 38772371

[34] K. Shi , D. C. Tian , Z. G. Li , A. F. Ducruet , M. T. Lawton , and F. D. Shi , “Global Brain Inflammation in Stroke,” Lancet Neurology 18, no. 11 (2019): 1058–1066, 10.1016/s1474-4422(19)30078-x. 31296369

[35] X. Lan , X. Han , Q. Li , Q. W. Yang , and J. Wang , “Modulators of Microglial Activation and Polarization After Intracerebral Haemorrhage,” Nature Reviews. Neurology 13, no. 7 (2017): 420–433, 10.1038/nrneurol.2017.69. 28524175 PMC5575938

[36] F. Gu , Z. Wang , H. Ding , et al., “Microglial Mitochondrial DNA Release Contributes to Neuroinflammation After Intracerebral Hemorrhage Through Activating AIM2 Inflammasome,” Experimental Neurology 382 (2024): 114950, 10.1016/j.expneurol.2024.114950. 39278588

[37] L. Kong , W. Li , E. Chang , et al., “mtDNA‐STING Axis Mediates Microglial Polarization via IRF3/NF‐κB Signaling After Ischemic Stroke,” Frontiers in Immunology 13 (2022): 860977, 10.3389/fimmu.2022.860977. 35450066 PMC9017276

[38] L. Song , H. Shen , F. Hong , W. Zhang , and H. Lu , “RORα‐Activated Mitophagy Attenuating Hypoxic‐Ischemic Encephalopathy via Suppression of Microglial cGAS‐STING Axis,” Frontiers in Immunology 16 (2025): 1592737, 10.3389/fimmu.2025.1592737. 40799648 PMC12341001

[39] S. J. Oh , J. Y. Lim , M. K. Son , et al., “TRPV1 Inhibition Overcomes Cisplatin Resistance by Blocking Autophagy‐Mediated Hyperactivation of EGFR Signaling Pathway,” Nature Communications 14, no. 1 (2023): 2691, 10.1038/s41467-023-38318-7. PMC10172196 37165076

[40] M. Durocher , B. P. Ander , G. Jickling , et al., “Inflammatory, Regulatory, and Autophagy Co‐Expression Modules and Hub Genes Underlie the Peripheral Immune Response to Human Intracerebral Hemorrhage,” Journal of Neuroinflammation 16, no. 1 (2019): 56, 10.1186/s12974-019-1433-4. 30836997 PMC6399982

[41] J. M. Park , D. H. Lee , and D. H. Kim , “Redefining the Role of AMPK in Autophagy and the Energy Stress Response,” Nature Communications 14, no. 1 (2023): 2994, 10.1038/s41467-023-38401-z. PMC10209092 37225695

[42] J. C. Stark , E. Wallace , R. Lim , and B. Leaw , “Characterization and Isolation of Mouse Primary Microglia by Density Gradient Centrifugation,” Journal of Visualized Experiments 132 (2018): 57065, 10.3791/57065. PMC5931276 29553508

[43] E. Mezey , Z. E. Tóth , D. N. Cortright , et al., “Distribution of mRNA for Vanilloid Receptor Subtype 1 (VR1), and VR1‐Like Immunoreactivity, in the Central Nervous System of the Rat and Human,” Proceedings of the National Academy of Sciences of the United States of America 97, no. 7 (2000): 3655–3660, 10.1073/pnas.97.7.3655. 10725386 PMC16295

[44] T. Sasamura , M. Sasaki , C. Tohda , and Y. Kuraishi , “Existence of Capsaicin‐Sensitive Glutamatergic Terminals in Rat Hypothalamus,” NeuroReport 9, no. 9 (1998): 2045–2048, 10.1097/00001756-199806220-00025. 9674591

[45] J. F. Sanchez , J. E. Krause , and D. N. Cortright , “The Distribution and Regulation of Vanilloid Receptor VR1 and VR1 5′ Splice Variant RNA Expression in Rat,” Neuroscience 107, no. 3 (2001): 373–381, 10.1016/s0306-4522(01)00373-6. 11718993

[46] L. Cristino , L. de Petrocellis , G. Pryce , D. Baker , V. Guglielmotti , and V. Di Marzo , “Immunohistochemical Localization of Cannabinoid Type 1 and Vanilloid Transient Receptor Potential Vanilloid Type 1 Receptors in the Mouse Brain,” Neuroscience 139, no. 4 (2006): 1405–1415, 10.1016/j.neuroscience.2006.02.074. 16603318

[47] A. Tóth , J. Boczán , N. Kedei , et al., “Expression and Distribution of Vanilloid Receptor 1 (TRPV1) in the Adult Rat Brain,” Brain Research. Molecular Brain Research 135, no. 1–2 (2005): 162–168, 10.1016/j.molbrainres.2004.12.003. 15857679

[48] X. Sha , J. Lin , K. Wu , J. Lu , and Z. Yu , “The TRPV1‐PKM2‐SREBP1 Axis Maintains Microglial Lipid Homeostasis in Alzheimer's Disease,” Cell Death & Disease 16, no. 1 (2025): 14, 10.1038/s41419-024-07328-8. 39809738 PMC11732990

[49] J. Zhang , K. Liu , O. Elmadhoun , et al., “Synergistically Induced Hypothermia and Enhanced Neuroprotection by Pharmacological and Physical Approaches in Stroke,” Aging and Disease 9, no. 4 (2018): 578–589, 10.14336/ad.2017.0817. 30090648 PMC6065296

[50] M. Muzzi , R. Felici , L. Cavone , et al., “Ischemic Neuroprotection by TRPV1 Receptor‐Induced Hypothermia,” Journal of Cerebral Blood Flow and Metabolism 32, no. 6 (2012): 978–982, 10.1038/jcbfm.2012.36. 22434066 PMC3367226

[51] J. Miyanohara , H. Shirakawa , K. Sanpei , T. Nakagawa , and S. Kaneko , “A Pathophysiological Role of TRPV1 in Ischemic Injury After Transient Focal Cerebral Ischemia in Mice,” Biochemical and Biophysical Research Communications 467, no. 3 (2015): 478–483, 10.1016/j.bbrc.2015.10.027. 26456642

[52] H. Akabori , H. Yamamoto , H. Tsuchihashi , et al., “Transient Receptor Potential Vanilloid 1 Antagonist, Capsazepine, Improves Survival in a Rat Hemorrhagic Shock Model,” Annals of Surgery 245, no. 6 (2007): 964–970, 10.1097/01.sla.0000255577.80800.e1. 17522523 PMC1876964

[53] K. Zhang , Z. Qin , J. Chen , et al., “TRPV1 Modulated NLRP3 Inflammasome Activation via Calcium in Experimental Subarachnoid Hemorrhage,” Aging (Albany NY) 16, no. 2 (2024): 1096–1110, 10.18632/aging.205379. 38180747 PMC10866436

[54] F. Corrigan , K. A. Mander , A. V. Leonard , and R. Vink , “Neurogenic Inflammation After Traumatic Brain Injury and Its Potentiation of Classical Inflammation,” Journal of Neuroinflammation 13, no. 1 (2016): 264, 10.1186/s12974-016-0738-9. 27724914 PMC5057243

[55] R. Rezzani , G. Favero , M. Gianò , et al., “Transient Receptor Potential Channels in the Healthy and Diseased Blood–Brain Barrier,” Journal of Histochemistry and Cytochemistry 72, no. 4 (2024): 199–231, 10.1369/00221554241246032. 38590114 PMC11020746

[56] D. X. Yang , Y. Jing , Y. L. Liu , et al., “Inhibition of Transient Receptor Potential Vanilloid 1 Attenuates Blood–Brain Barrier Disruption After Traumatic Brain Injury in Mice,” Journal of Neurotrauma 36, no. 8 (2019): 1279–1290, 10.1089/neu.2018.5942. 30351220

[57] H. E. Gibson , J. G. Edwards , R. S. Page , M. J. Van Hook , and J. A. Kauer , “TRPV1 Channels Mediate Long‐Term Depression at Synapses on Hippocampal Interneurons,” Neuron 57, no. 5 (2008): 746–759, 10.1016/j.neuron.2007.12.027. 18341994 PMC2698707

[58] A. E. Chávez , C. Q. Chiu , and P. E. Castillo , “TRPV1 Activation by Endogenous Anandamide Triggers Postsynaptic Long‐Term Depression in Dentate Gyrus,” Nature Neuroscience 13, no. 12 (2010): 1511–1518, 10.1038/nn.2684. 21076423 PMC3058928

[59] P. Muñoz , A. Aschrafi , and P. R. Moya , “Connecting Synaptic Activity With Plasticity‐Related Gene Expression: From Molecular Mechanisms to Neurological Disorders,” Neural Plasticity 2016 (2016): 7149527, 10.1155/2016/7149527. 27088015 PMC4818811

[60] Z. Gan‐Or , P. A. Dion , and G. A. Rouleau , “Genetic Perspective on the Role of the Autophagy‐Lysosome Pathway in Parkinson Disease,” Autophagy 11, no. 9 (2015): 1443–1457, 10.1080/15548627.2015.1067364. 26207393 PMC4590678

[61] P. Wang , B. Z. Shao , Z. Deng , S. Chen , Z. Yue , and C. Y. Miao , “Autophagy in Ischemic Stroke,” Progress in Neurobiology 163‐164 (2018): 98–117, 10.1016/j.pneurobio.2018.01.001. 29331396

[62] W. Gao , Y. Sun , M. Cai , et al., “Copper Sulfide Nanoparticles as a Photothermal Switch for TRPV1 Signaling to Attenuate Atherosclerosis,” Nature Communications 9, no. 1 (2018): 231, 10.1038/s41467-017-02657-z. PMC5768725 29335450

[63] L. Q. Zhou , M. H. Dong , Z. W. Hu , et al., “Staged Suppression of Microglial Autophagy Facilitates Regeneration in CNS Demyelination by Enhancing the Production of Linoleic Acid,” Proceedings of the National Academy of Sciences of the United States of America 120, no. 1 (2023): e2209990120, 10.1073/pnas.2209990120. 36577069 PMC9910603

[64] R. Berglund , A. O. Guerreiro‐Cacais , M. Z. Adzemovic , et al., “Microglial Autophagy‐Associated Phagocytosis Is Essential for Recovery From Neuroinflammation,” Science Immunology 5, no. 52 (2020): eabb5077, 10.1126/sciimmunol.abb5077. 33067381

[65] N. A. H. Brooks , I. Riar , and A. Klegeris , “Mitochondrial Damage‐Associated Molecular Patterns: Neuroimmunomodulators in Central Nervous System Pathophysiology,” Neural Regeneration Research 21, no. 4 (2026): 1322–1338, 10.4103/nrr.Nrr-d-24-01459. 40537002 PMC12407514

[66] D. P. Mohapatra and C. Nau , “Regulation of Ca2+−Dependent Desensitization in the Vanilloid Receptor TRPV1 by Calcineurin and cAMP‐Dependent Protein Kinase,” Journal of Biological Chemistry 280, no. 14 (2005): 13424–13432, 10.1074/jbc.M410917200. 15691846

[67] J. Jung , J. S. Shin , S. Y. Lee , et al., “Phosphorylation of Vanilloid Receptor 1 by Ca2+/Calmodulin‐Dependent Kinase II Regulates Its Vanilloid Binding,” Journal of Biological Chemistry 279, no. 8 (2004): 7048–7054, 10.1074/jbc.M311448200. 14630912

[68] S. Y. Lee , J. H. Lee , K. K. Kang , S. Y. Hwang , K. D. Choi , and U. Oh , “Sensitization of Vanilloid Receptor Involves an Increase in the Phosphorylated Form of the Channel,” Archives of Pharmacal Research 28, no. 4 (2005): 405–412, 10.1007/bf02977669. 15918513

[69] M. Studer and P. A. McNaughton , “Modulation of Single‐Channel Properties of TRPV1 by Phosphorylation,” Journal of Physiology 588, no. Pt 19 (2010): 3743–3756, 10.1113/jphysiol.2010.190611. 20693293 PMC2998224

[70] N. A. Veldhuis , D. P. Poole , M. Grace , P. McIntyre , and N. W. Bunnett , “The G Protein‐Coupled Receptor‐Transient Receptor Potential Channel Axis: Molecular Insights for Targeting Disorders of Sensation and Inflammation,” Pharmacological Reviews 67, no. 1 (2015): 36–73, 10.1124/pr.114.009555. 25361914

[71] G. Gao , S. Nakamura , S. Asaba , Y. Miyata , H. Nakayama , and T. Matsui , “Hesperidin Preferentially Stimulates Transient Receptor Potential Vanilloid 1, Leading to NO Production and Mas Receptor Expression in Human Umbilical Vein Endothelial Cells,” Journal of Agricultural and Food Chemistry 70, no. 36 (2022): 11290–11300, 10.1021/acs.jafc.2c04045. 36039965

