# Supplementary material for: Inhibition of Microglial TRPV1 Ameliorates Brain Injury After Intracerebral Hemorrhage by Suppressing AMPK/PINK1‐Mediated Mitophagy
Source: CNS Neurosci Ther. 2026 Apr 20;32(4):e70881. doi: 10.1002/cns.70881 (PMC13093729; doi:10.1002/cns.70881)
Supplement: Supplementary file 4 — Figure S1: Schematic diagram of animal experiment design. Figure S2: The effects of different doses of CAP and CPZ on mNSS scores in ICH mice. Figure S3: The effects of CPZ on BBB in ICH mice. Figure S4: CPZ exerts neuroprotective effects through microglial TRPV1 after ICH. Figure S5: BV2 conditioned medium was associated with HT22 survival. Figure S6: TRPV1 regulated mitophagy and endocytosis in BV2 cells. [file CNS-32-e70881-s004.docx]

**Supplementary materials**

**Inhibition of microglial TRPV1 ameliorates brain injury after intracerebral hemorrhage by suppressing AMPK/PINK1-mediated mitophagy**

Fig. S1 Schematic Diagram of Animal Experiment Design

Experiment 1: Temporal and Spatial Expression of TRPV1 after ICH. Mice were allocated into control, 48-hour, 72-hour (3-day), and 1-week post-ICH groups. TRPV1 protein levels in striatal tissues were assessed by Western blot (n=6). Immunofluorescence (n=6) was performed at 3 days post-ICH to determine TRPV1 localization.

Experiment 2: Effect of TRPV1 Modulation on ICH Pathology. Mice received intraperitoneal injections of vehicle, CPZ (TRPV1 inhibitor), or CAP (TRPV1 agonist) post-ICH. Hematoma volume, hemoglobin and brain water content were measured on brain at day 3 (n=6/group). MRI was performed at days 1, 3, and 7 (n=6/group). Immunofluorescence for FJC and TUNEL (n=6/group) and Nissl staining (n=4/group) were conducted at day 3. Nissl staining (n=4/group) were conducted at day 28.

Experiment 3: Effect of TRPV1 Inhibition on Neurological Function. ICH mice were treated with vehicle or CPZ. Behavioral tests (mNSS, forelimb placement, corner turn) were performed pre-ICH and at days 1, 3, and 7 post-ICH. Open field test and Morris water maze tests were conducted at days 14 and 28, respectively (n=6/group).

Experiment 4: TRPV1 in Autophagy and Mitochondrial Dysfunction. ICH mice received vehicle or CPZ. Immunofluorescence for BBB integrity, microglial polarization, and autophagy markers (n=6/group), TEM for mitochondrial ultrastructure (n=6/group), and Western blot for autophagy-related proteins (n=6/group) were analyzed at day 3 post-ICH.

Experiment 5: Microglial Depletion and TRPV1 Inhibition. Mice were fed PLX5622 chow for 3 weeks pre-ICH, then treated with vehicle or CPZ. Apoptosis was assessed by immunofluorescence at day 3 post-ICH (n=6/group).

Experiment 6: Microglia-Specific TRPV1 Knockout in ICH. Microglia-specific TRPV1 cKO mice (TRPV1flox/flox; Cx3cr1Cre) and controls (TRPV1flox/flox) received intraperitoneal injections of tamoxifen (75 mg/kg/day) for five consecutive days and were used for experiments 2 weeks later. Apoptosis was evaluated by immunofluorescence (n=6/group), and behavioral tests were performed as in Experiment 3 (n=6/group).

Fig. S2

The effects of different doses of CAP and CPZ on mNSS scores in ICH mice. The mNSS tests were conducted one week after ICH. The doses of CAP and CPZ are indicated in mg/kg within parentheses. For this test, 4 to 6 mice were used in each group. *P<0.05, *P<0.01, CAP vs. Vehicle; #P<0.05, CPZ vs. Vehicle

Fig. S3

The effects of CPZ on BBB in ICH mice. (a-b) Images of co-staining for ZO-1 (red) and vascular endothelial cells (CD31, green) three days after ICH and results of quantitative analysis. (c-d) Images of co-staining for claudin5 (red) and CD31(green) and results of quantitative analysis. ***P<0.001 vs. Vehicle. n=6/group. Scale bar=100 μm.

Fig. S4

CPZ exerts neuroprotective effects through microglial TRPV1 after ICH. (a) Representative immunofluorescence images showing colocalization of TRPV1 (green) with the neuronal marker NeuN (red) in the perihematomal area 3 days post-ICH. Scale bar = 20 μm. (b) Representative images of TUNEL (green) and NeuN (red) costaining in the perihematomal area of microglia-specific TRPV1 conditional knockout (cKO) mice 3 days after ICH, with or without CPZ treatment. Scale bar = 100 μm. (c) Quantitative analysis of apoptotic neurons (TUNEL⁺NeuN⁺) in (b). (d) Representative images of autophagic vesicles (LC3B, green) costained with mitochondria (VDAC, red) in the perihematomal area of cKO mice 3 days post-ICH, with or without CPZ treatment. (e) Quantitative analysis of Mander's colocalization coefficient for LC3B with VDAC in (d). (f) Representative images of mitophagy marker Parkin (green) costained with mitochondria (VDAC, red) in the perihematomal area of cKO mice 3 days post-ICH, with or without CPZ treatment. (g) Quantitative analysis of Mander's colocalization coefficient for Parkin with VDAC in (f). Scale bar is 30 μm in d and f, scale bar is 10 μm in ZOOM images of d and f. Data are presented as mean±SD. ns, not significant. n = 5 mice per group.

Fig. S5

BV2 conditioned medium was associated with HT22 survival. (a) Experimental workflow for using BV2 conditioned medium to culture HT22 cells. Created with BioRender.com. (b) Images of JC-1 staining in HT22 cells, indicating mitochondrial membrane potential. Scale bar=30 μm. (c) Quantification of JC-1 red/green fluorescence ratio in (b). (d) Representative images of caspase-3 (green) and NeuN (red) costaining in HT22 neurons cultured with conditioned medium from BV2 cells under indicated conditions. Scale bar = 20 μm. (e) Quantitative analysis of apoptotic neurons (caspase-3⁺NeuN⁺) in (d). (f) ELISA of IL-1β levels in BV2-conditioned medium under indicated conditions. (g) ELISA of TNF-α levels in BV2-conditioned medium under indicated conditions. (h) Representative images of PicoGreen staining (green) showing double-stranded DNA localization in BV2 cells under indicated conditions. Arrows indicate cytosolic mtDNA release. Nuclei were counterstained with DAPI (blue). Scale bar = 10 μm. *P < 0.05, **P < 0.01, ***P < 0.001, ***P < 0.001 vs. Hb + DMSO group. n=6/group. (i-j) Images of autophagic vesicles (LC3B, green) costained with mitochondria (mitoTracker, red) and quantitative analysis of mitophagosomes in HT22 cells treated with BV2 conditioned medium. (k-l) Images of mitophagy markers (Parkin, green) costained with mitochondria (mitoTracker, red) and quantitative analysis of Mander's coefficient for Parkin with mitoTracker in HT22 cells. **P<0.01, ***P<0.001 vs. Vehicle, n=5/group in i, n=4/group in j. Scale bar=30 μm.

Fig. S6

TRPV1 regulated mitophagy and endocytosis in BV2 cells. (a) Representative Western blot images showing PINK1, Parkin, and mitochondrial marker COX IV in the mitochondrial fraction of BV2 cells under indicated conditions. (b) Quantitative analysis of relative PINK1 and Parkin protein levels in the mitochondrial fraction after 24 h of Hb treatment, with or without CPZ. (c) Representative Western blot images showing PINK1, Parkin, and cytosolic marker Actin in the cytosolic fraction of BV2 cells under indicated conditions. (d) Quantitative analysis of relative PINK1 and Parkin protein levels in the cytosolic fraction after 24 h of Hb treatment, with or without CPZ. (e) Representative images of PKH26-labeled red blood cell phagocytosis by BV2 cells under indicated conditions. Scale bar = 10 μm. (f-g) Quantitative analysis of the percentage of BV2 cells phagocytosing PKH26-labeled red blood cells and the relative number of phagocytosed PKH26-labeled red blood cells per BV2 cell in (e). Data are presented as mean±SD. *P < 0.05, **P < 0.01, ***P < 0.001, ****P < 0.0001 vs. Vehicle; n=6/group. (h) Western blot images showing downregulation of TRPV1 by siRNA. (i) Quantitative analysis of TRPV1 protein expression levels after siRNA application. **P<0.01, ***P<0.001 vs. siNC, n=6/group. (j) Images of autophagic vesicles (LC3B, green) costained with mitochondria (mitoTracker, red) before and after siRNA intervention in Hb-treated cells. (k) Quantitative analysis of mitochondrial autophagic vesicles after siRNA intervention. Scale bar is 30 μm, scale bar is 10 μm in ZOOM images. **P<0.01, ***P<0.001 vs. siNC+Hb, n=6/group.
